# Supplementary material for: Protocol for a prospective, controlled, cross-sectional, diagnostic accuracy study to evaluate the specificity and sensitivity of ambulatory monitoring systems in the prompt detection of hypoxia and during movement
Source: BMJ Open. 2020 Jan 12;10(1):e034404. doi: 10.1136/bmjopen-2019-034404 (PMC7044954; doi:10.1136/bmjopen-2019-034404)
Supplement: Supplementary data [file bmjopen-2019-034404supp001.pdf]

Appendix 1: Wearable Ambulatory Monitors Summary

Monitors	Vital signs	Application	Monitoring method	Data storage	
**VitalPatch®**<br><br>**CE marked**	Respiratory rate (rpm), Heart rate (bpm)	Chest worn	1-lead ECG and accelerometer signals are used for the accurate estimation of heart rate and respiratory rate.	Data collected in real time when the device is synchronised via BLE with vHDU app, otherwise the data are recorded in the device's memory, and downloaded afterwards via the vHDU app.	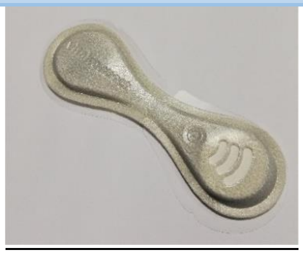
**CheckMe™ O2+**<br><br>**CE Marked**	SpO2 (%),<br>Pulse rate (bpm)	Wrist worn with thumb ring probe.	Transmittance PPG is used to estimate SpO2. The Infrared PPG is used to estimate pulse rate.	Data collected in real time when the device is synchronised via BLE with vHDU app.	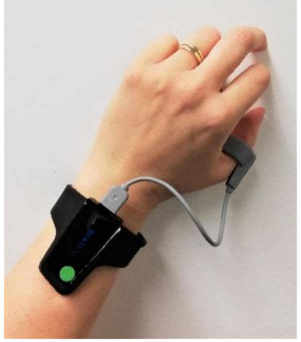

**AP-20®**<br><br>**CE Marked**	SpO2 (%),<br>Pulse rate (bpm),<br>Respiratory rate (rpm).	Wrist worn with finger-tip sensor. Includes Nasal flow sensor.	Transmittance PPG is used to estimate SpO2. The Infrared PPG is used to estimate pulse rate.<br><br>The airflow signal is used to determine the Respiratory Rate	Data collected in real time when the device is synchronised via BLE with vHDU app.<br><br>All data are also stored in the device memory, and downloaded afterwards via a the AP-20® software.	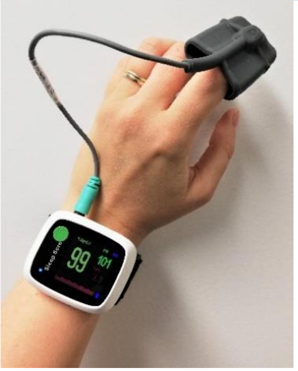
**Wavelet**<br><br>**No regulatory approval at the time of the study**	SpO2 (%),<br>Pulse rate (bpm)	Wrist worn	Reflectance PPG is used to estimate SpO2. The Infrared PPG is used to estimate pulse rate.<br><br>Accelerometer data is used to discard estimations perturbed by moment.	Wavelet onsite App is used to collect the data.<br><br>De-identifiable Data will be transferred to the Cloud storage but data may remain on the storage system even after being downloaded by the research team<br><br>Access to storage data is as per Cloud licensing agreement.	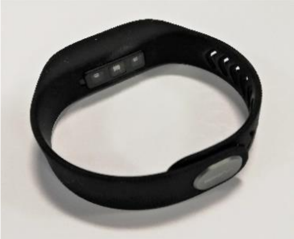
**<u>WristOX2</u> 3150 OEM BLE**<br><br><br>**FDA Approved**	SpO2 (%),<br>Pulse rate (bpm)	Wrist worn with finger-tip sensor.	Transmittance PPG is used to estimate SpO2. The Infrared PPG is used to estimate pulse rate.	Data collected in real time when the device is synchronised via BLE with vHDU app.	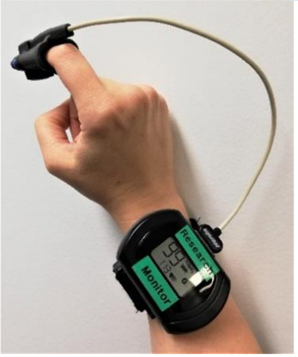
