# Supplementary material for: Protocol for a prospective, controlled, cross-sectional, diagnostic accuracy study to evaluate the specificity and sensitivity of ambulatory monitoring systems in the prompt detection of hypoxia and during movement
Source: BMJ Open. 2020 Jan 12;10(1):e034404. doi: 10.1136/bmjopen-2019-034404 (PMC7044954; doi:10.1136/bmjopen-2019-034404)
Supplement: Supplementary data [file bmjopen-2019-034404supp002.pdf]

Appendix 2: Functional Movement Testing – Tablet Protocol

Press home
Type in code _ _ _ _
Go to safari (compass icon)
Search 'Google'
Search 'Oxford weather forecast'
Select BBC weather
Scroll across hourly forecast and select 'see more weather for ______'
Scroll across again
Scroll down the page and select and play the BBC South Today weather video.
Press the full screen button in the right bottom corner of the screen. Watch the video then minimise the screen.
Go to the internet search bar and search 'google'
Search 'Amazon'
Select the Amazon website
Search 'Bicycle' and scroll to select a bicycle you like
Add to basket, then navigate back to the search page
Search 'Bicycle Helmet' and again scroll and select one.
Press the back button in the top left corner until back to the safari/google page
Type following sums:
100 x 70 / 45 =
80 + 6 + 95 + 43 + 51 + 15 =
