# Supplementary material for: Protocol for a prospective, controlled, cross-sectional, diagnostic accuracy study to evaluate the specificity and sensitivity of ambulatory monitoring systems in the prompt detection of hypoxia and during movement
Source: BMJ Open. 2020 Jan 12;10(1):e034404. doi: 10.1136/bmjopen-2019-034404 (PMC7044954; doi:10.1136/bmjopen-2019-034404)
Supplement: Supplementary data [file bmjopen-2019-034404supp003.pdf]

Appendix 3: Model Consent Form

Study Code: *Sub-Study code:* *Participant identification number:*

**V**	**H**	**D**	**U**	—	H	—			

CONSENT FORM

Virtual HDU: Hypoxia Study. Accuracy and validity testing of ambulatory monitoring system.

Name of Researcher: *If you agree, please initial box*

1. I confirm that I have read and understood the information sheet dated 17/JUN/2019 version 4.0 for the above study I have had the opportunity to consider the information, ask questions and have had these answered satisfactorily.	
2. I understand that my participation is voluntary and that I am free to withdraw at any time without giving any reason, without my medical care or legal rights being affected.	
3. I understand who will have access to personal data provided, how the data will be stored and what will happen to the data at the end of the project.	
4. I understand and consent for all required screening tests that will include medical history review, breathing tests, urine pregnancy test (female participants only) and blood sample tests.	
5. I agree to physiological vital sign monitoring with the use of ambulatory monitoring device(s).	
6. I agree to cannulation (insertion of tube in your radial artery) and hypoxic exposure (controlled reduction of oxygen levels) for the duration of the testing phase of the study and understand these procedures and potential (although very rare) complications.	
7. I agree to donate up to 15(teaspoon-sized) blood samples. I consider these samples a gift to the University of Oxford and I understand I will not gain any direct personal or financial benefit from them. I also understand these will be discarded and not retained by the research team after the study visit.	
8. I understand that in some cases the monitoring systems being used require initial upload of vital signs data to their proprietary Cloud storage facility that might be abroad, from which these data are then downloaded. I understand in this case this will be discussed with me beforehand and no identifiable data will be included in this upload.	
9. I understand how to raise a concern and make a complaint.	
**10. I agree to take part in this study.**	

______________________ ________________ __________________________

Name of Participant *Date* *Signature*

______________________ ________________ __________________________

Name of Person taking Consent *Date* *Signature*

**1 copy for participant; 1 copy for researcher site file; 1 (original) to be kept in medical notes* (if participant is a patient).
