# Supplementary material for: Protocol for a prospective, controlled, cross-sectional, diagnostic accuracy study to evaluate the specificity and sensitivity of ambulatory monitoring systems in the prompt detection of hypoxia and during movement
Source: BMJ Open. 2020 Jan 12;10(1):e034404. doi: 10.1136/bmjopen-2019-034404 (PMC7044954; doi:10.1136/bmjopen-2019-034404)
Supplement: Author's manuscript [file bmjopen-2019-034404.draft_revisions.pdf]

BMJ Open

BMJ Open is committed to open peer review. As part of this commitment we make the peer review history of every article we publish publicly available.

When an article is published we post the peer reviewers' comments and the authors' responses online. We also post the versions of the paper that were used during peer review. These are the versions that the peer review comments apply to.

The versions of the paper that follow are the versions that were submitted during the peer review process. They are not the versions of record or the final published versions. They should not be cited or distributed as the published version of this manuscript.

BMJ Open is an open access journal and the full, final, typeset and author-corrected version of record of the manuscript is available on our site with no access controls, subscription charges or pay-per-view fees (http://bmjopen.bmj.com).

If you have any questions on BMJ Open's open peer review process please email info.bmjopen@bmj.com

BMJ Open

Protocol for a prospective, controlled, cross-sectional, diagnostic accuracy study to evaluate the specificity and sensitivity of ambulatory monitoring systems in the prompt detection of hypoxia and during movement.

Journal:	*BMJ Open*
Manuscript ID	bmjopen-2019-034404
Article Type:	Protocol
Date Submitted by the Author:	20-Sep-2019
Complete List of Authors:	Areia, Carlos; University of Oxford, Nuffield Department of Clinical Neurosciences<br>Vollam, Sarah; University of Oxford, Nuffield Department of Clinical Neurosciences<br>Piper, Philippa; Oxford University Hospitals NHS Foundation Trust, Adult Intensive Care Unit<br>King, Elizabeth; Oxford University Hospitals NHS Foundation Trust, Adult Intensive Care Unit<br>Ede, Jody; University of Oxford, Nuffield Department of Clinical Neurosciences<br>Young, Louise; University of Oxford, Nuffield Department of Clinical Neurosciences<br>Santos, Mauro; University of Oxford, Institute of Biomedical Engineering, Department of Engineering Science<br>Pimentel, Marco; University of Oxford, Institute of Biomedical Engineering, Department of Engineering Science<br>Roman, Cristian; University of Oxford, Institute of Biomedical Engineering, Department of Engineering Science<br>Harford, Mirae; University of Oxford, Nuffield Department of Clinical Neurosciences<br>Shah, Akshay; University of Oxford, Radcliffe Department of Medicine<br>Gustafson, Owen; Oxford University Hospitals NHS Foundation Trust, Adult Intensive Care Unit<br>Rowland, Matthew; University of Oxford, Nuffield Department of Clinical Neurosciences<br>Tarassenko, Lionel; University of Oxford, Institute of Biomedical Engineering, Department of Engineering Science<br>Watkinson, Peter; University of Oxford, Nuffield Department of Clinical Neurosciences
Keywords:	Adult intensive & critical care < ANAESTHETICS, INTENSIVE & CRITICAL CARE, Health informatics < BIOTECHNOLOGY & BIOINFORMATICS, BIOTECHNOLOGY & BIOINFORMATICS

SCHOLARONE™
Manuscripts

Protocol for a prospective, controlled, cross-sectional, diagnostic accuracy study to evaluate the specificity and sensitivity of ambulatory monitoring systems in the prompt detection of hypoxia and during movement.

Carlos Areia[1], Sarah Vollam[1], Philippa Piper[2], Elizabeth King[2], Jody Ede[1], Louise Young[1], Mauro Santos[3], Marco Pimentel[3], Cristian Roman[3], Mirae Harford[1], Akshay Shah[4], Owen Gustafson[2], Matthew Rowland[1], Lionel Tarassenko[3], Peter Watkinson[1]

1 – Critical Care Research Group, Nuffield Department of Clinical Neurosciences, University of Oxford, Oxford, United Kingdom
2 – Adult Intensive Care Unit, Oxford University Hospitals NHS Foundation Trust, Oxford, United Kingdom
3 – Institute of Biomedical Engineering, Department of Engineering Science, University of Oxford, Oxford, United Kingdom
4 – Radcliffe Department of Medicine, University of Oxford, Oxford, United Kingdom

Corresponding author:
Carlos Areia,
Critical Care Research Group,
Kadoorie Centre, Level 3 John Radcliffe Hospital
Oxford, United Kingdom.
OX3 9DU

carlos.morgadoareia@ndcn.ox.ac.uk

For peer review only

ABSTRACT

Introduction: Automated continuous ambulatory monitoring may provide an alternative to intermittent manual vital signs monitoring. This has the potential to improve frequency of measurements, timely escalation of care and patient safety. However, a major barrier to the implementation of these wearable devices in the ward environment is their uncertain reliability, efficiency and data fidelity. The purpose of this study is to test performance of selected devices in a simulated clinical setting including during movement and low levels of peripheral oxygen saturation.

Methods and Analysis: This is a single centre, prospective, controlled, cross-sectional, diagnostic accuracy study to determine the specificity and sensitivity of currently available ambulatory vital signs monitoring equipment in the detection of hypoxia and the effect of movement on data acquisition. We will recruit up to 45 healthy volunteers that will attend a single study visit; starting with a movement phase and followed by the hypoxia exposure phase where we will gradually decrease saturation levels down to 80%. We will simultaneously test one chest patch, one wrist worn only and three wrist worn with finger probe devices against 'clinical standard 'and 'gold standard' references. We will measure peripheral oxygen saturations, pulse rate, heart rate and respiratory rate continuously and arterial blood gases intermittently throughout the study.

Ethics and Dissemination: This study has received ethical approval by the East of Scotland Research Ethics Service REC 2 (19/ES/0008). The results will be broadly distributed through conference presentations and peer-reviewed publications.

Keywords: Hypoxia, ambulatory monitoring, vital signs, wearable devices

ARTICLE SUMMARY

Strengths and Limitations of this study

- Controlled hypoxia exposure in a standardised environment for all participants
- Outcome comparison to both clinical and gold standards
- Largest study in healthy volunteers
- Once specificity and sensitivity have been established in healthy volunteers; devices will be tested in the target hospital population

Word count: 4057

Protocol Version: 1.0
Protocol Date: 07 Aug 2019
Public Title: Testing the effects of low levels of blood oxygen and movement in the accuracy of wearable vital signs monitors
Trial Registration number: ISRCTN61535692 registered on 10/06/2019
Sponsorship: University of Oxford. Clinical Trials & Research Governance (ctrg@admin.ox.ac.uk)
Funding: This study/project is funded by the NIHR Biomedical Research Centre, Oxford. PW and LT are supported by the NIHR Biomedical Research Centre, Oxford.
Disclaimer: The views expressed are those of the authors and not necessarily those of the NIHR or the Department of Health and Social Care.
Recruitment start date: 18 Jun 2019
Status: Recruiting

For peer review only

INTRODUCTION

Failure to recognise and act on physiological indicators of worsening acute illness in hospital wards is a prevalent problem recognised over twenty years ago [1]. Current practice involves the use of early warning scoring systems, which monitor standard vital signs. These include intermittent measurements of pulse rate, respiratory rate, blood pressure, oxygen saturations and temperature. The frequency of vital signs measurements is usually guided by the clinical condition of the patient.

Intermittent measurement of these vital signs can be time consuming for healthcare professionals [2] and therefore the desired frequency of observations is often not achieved [3]. Infrequent measurement of vital signs may also miss clinical deteriorations between these measurements [4]. Thus, more sustainable, accurate and less time-consuming monitoring methods would be highly desirable.

Wearable ambulatory monitors (AM) may provide an alternative to intermittent vital signs monitoring by enabling the continuous monitoring of vital signs parameters. In addition to reducing the burden of intermittent measurement of vital signs on staff, continuous monitoring has the potential to facilitate earlier detection of deranged physiological parameters [5]. A major barrier to the clinical implementation of these wearable devices is their uncertain reliability, efficiency and data fidelity [6]. In particular, the effect of motion on its accuracy is under-investigated. Recent work by Louie et al (2018) tested four non-ambulatory pulse oximeters and found that motion impaired performance throughout a clinically relevant range of measurements. Less accuracy was reported at lower arterial oxygen saturations, which is undesirable in clinical practice [7].

This study is part of the Virtual High Dependency Unit (vHDU) project, a collaboration between the Institute of Biomedical Engineering and clinicians from the Nuffield Department of clinical Neurosciences at the University of Oxford. This is a phased project aiming to refine and integrate ambulatory monitoring systems for use in clinical practice. Previous phases have tested device wearability and in situ testing on hospital wards. The purpose of this study is to test the performance of selected devices in a simulated clinical setting which will involve participant movement and inducing low peripheral oxygen saturations.

METHODS

This protocol follows the “Standard Protocol Items: Recommendations for Interventional Trials” (SPIRIT) reporting guidelines [8]. We registered this protocol in a public database: ISRCTN61535692.

Study objectives

Primary objective: To determine the specificity and sensitivity of currently available ambulatory vital signs monitoring equipment for the detection of hypoxia.

Secondary objective: To determine the effect of movement on data acquisition by currently available ambulatory vital signs monitoring equipment.

These objectives will be assessed by comparing continuous peripheral oxygen saturations, pulse rate, heart rate and respiratory rate data from each ambulatory monitor with arterial blood oxygen saturation measured through arterial blood sampling, pulse rate derived from the arterial blood signal, heart rate derived from standard care 3-lead ECG, and respiratory rate derived from capnography and manual counting.

Study Design

For peer review only

Prospective, observational cross-sectional cohort study. Vital signs parameters from study devices will be compared with 'gold standard' and 'clinical standard' measurements.

Sample size

Our sample size calculation is based on the ISO 80601-2-61:2019 guideline for pulse-oximetry equipment accuracy testing. This requires at least 200 data points balanced across each decadal range (70 - 80%, 80 - 90%, 90 - 100%) of the SaO2 range 70 – 100%, from at least 10 subjects. Up to 45 healthy adult subjects who meet the inclusion criteria will participate in the study. For the broadest application to the largest group of participants, the subjects should vary in their physical characteristics to the greatest extent possible.

Recruitment

Up to 45 healthy volunteers will be recruited, with adverts placed in appropriate target locations such as college common spaces and university buildings. The adverts will contain a description of the study and the number and email contacts of one of the members of the research team.

Inclusion criteria for the study are: willing and able to give informed consent for participation in the study; men and women aged 18 or over; and in generally good health.

Exclusion criteria are: allergies to adhesive dressings (such as bio-occlusive dressings or micropore) or local anaesthetic (e.g. lidocaine); intra-cardiac device (e.g. Permanent pacemaker) or previous wrist arterial line; epilepsy; angina, congenital heart disease or history of severe cardio-pulmonary disease; history of anaemia (reported in the pre-screening telephone call), haemoglobinopathy or haemoglobin below 100 g/l on first test; resting hypoxaemia (SpO2 <94%) or significant cardiopulmonary disease rendering exposure to alveolar hypoxia unsafe, as determined by the research physician; pregnancy or breast feeding; clotting disorders and use of antiplatelet or anticoagulant medication (such as aspirin); and claustrophobia precluding spell in the hypoxic exposure.

Study procedures

Initial Contact

Healthy volunteers will contact the research team via telephone/email to express their interest in the study. The research team will provide further information including the Participant Information Sheet (PIS). If volunteers wish to proceed, a telephone appointment will be arranged to complete a brief pre-screening assessment with a research nurse/physiotherapist (supported by a senior anaesthetist).

Pre-screening assessment

During the pre-screening telephone appointment, the study will be discussed further and general screening questions will be asked, to confirm eligibility. Questions will be encouraged to ensure the potential participant understands the study. If the potential participant agrees to take part, an appointment for the hypoxia exposure visit will be agreed.

Study visit

Screening Assessment

The screening assessment will be completed by an appropriately qualified, medically trained member of the research team, who will confirm eligibility for the hypoxic exposure phase. This will include a urinary pregnancy test for all female participants of child bearing potential. Pregnancy is an exclusion criteria for the study as the effects of hypoxia on pregnancy are unknown [9].

Arterial Line Insertion

For peer review only

After confirmation of eligibility, an arterial line will be inserted into the non-dominant radial artery of each participant on the day of the study visit under local anaesthesia.

Initial blood gas sampling

The first arterial blood gas (ABG) measurement will be assessed by the anaesthetist to confirm haemoglobin concentration ≥ 100 g/l. If the haemoglobin is below this level, the participant will be withdrawn from the study, the arterial line removed and the participant advised to discuss this finding with their general practitioner (as stated in the PIS).

Placement of devices

Participants will wear several ambulatory monitoring devices (AMD) which may include a chest worn patch (VitalPatch®), a purely wrist worn device (Wavelet) and up to 3 wrist worn devices with finger probe(CheckMe™ O2+, AP-20 and WristOX2 3150 BLE). The AMD detect various combinations of pulse oximetry, pulse rate, heart rate and respiratory rate (please refer to Appendix 2 for device details). Participants will be asked to wear a maximum of 5 study devices during the hypoxia exposure visit (Figure 1).

Finger probe position randomisation

Up to three study device finger probes will be worn, in addition to the 'clinical standard' reference bedside monitor finger probe. The CheckMeTM O2+ will always be worn on the thumb as per manufacturer recommendation [10]. To ensure parity of testing, the position of the other three finger probes on the 2nd, 3rd and 4th fingers will be randomised (using https://www.random.org/) per study visit day, ensuring an even distribution of placement, as per Table 1).

Table 1 – Device combinations

**Devices placement**	**Combination 1 (n=10)**	**Combination 2 (n=10)**	**Combination 3 (n=10)**
**Chest**	VitalPatch®	VitalPatch®	VitalPatch®
**Wrist**	Wavelet	Wavelet	Wavelet
**1st (thumb)**	CheckMe™ O2+	CheckMe™ O2+	CheckMe™ O2+
**2nd (index finger)**	Philips monitor (MX450)	WristOX2 3150 BLE	AP-20
**3rd (middle finger)**	AP-20	Philips monitor (MX450)	WristOX2 3150 BLE
**4th (ring finger)**	WristOX2 3150 BLE	AP-20	Philips monitor (MX450)

Stage 1 - Movement phase

During the movement phase participants will be seated in a chair and asked to complete a series of consecutive standardised movements, as detailed in Table 2. An ABG and manual (counted) respiratory rate will be taken at the end of each movement.

Table 2 - Standardised movements to be tested

**Movement**	**Task**
Standing from chair using arms to push up/sit down	20x repetitions
Tapping	Volunteer to tap a surface with the aid of a metronome set at 100 bpm for 2 minutes

For peer review only

Rubbing	Volunteer to complete a sideways rubbing movement with the aid of a metronome set at 100 bpm for 2 minutes
Drinking from plastic cup	20 x lift/drink/put down
Turning page	50x page turns
Using tablet	As per protocoled instructions (Appendix 1)

Stage 2 - Hypoxia Exposure phase

Participants will move to a bed and lie comfortably in a semi-recumbent, supine position (Figure 2). A tight-fitting silicone facemask will be placed and connected to a hypoxicator unit (Everest Summit Hypoxic Generator, www.altitudecentre.com). If required, additional 7% oxygen in nitrogen from a cylinder will be entrained into the hypoxicator circuit to ensure tight control of fraction of inspired oxygen (FiO_2) provided to the participant [11]. Inhaled FiO_2 will be monitored by an in-line gas analyser and end-tidal carbon dioxide ($etCO_2$) will be also recorded via capnography using the Philips monitor MX450 (www.philips.co.uk).

During the hypoxia exposure phase, oxygen saturations from the 'clinical standard' Philips monitor will guide the titration of the hypoxicator. 7% oxygen in nitrogen will be used to further lower FiO_2 if required. An ABG will be sampled when the participant reaches and remains stable at each pre-specified target peripheral oxygen saturation level (95%, 90%, 87%, 85%, 83%, 80%). We specified these saturations to allow assessment for our use case of prompt detection of hypoxia in normal adult patients in a ward environment, including multiple assessments within the 83-95% range, and one assessment at the top end of the 70-80% range, considered severe hypoxia.

Blood sampling

Up to 15 ABG samples will be taken. Samples will be discarded at the end of the laboratory session into clinical waste and no blood will be retained by the study.

At the end of the study visit the arterial line will be removed and firm pressure applied to the site until haemostasis is achieved. A sterile dressing will be applied and advice given to the participant on action to take if any bleeding occurs.

Facilities and Research Staff

Facilities

All study visits will occur in the Cardiovascular Clinical Research Facility, Level 1 Oxford Heart Centre, John Radcliffe Hospital, Headley Way, Headington, Oxford, OX3 9DU.

Roles and Responsibilities

Each study visit day will be staffed by one senior anaesthetist, four clinical researchers and one engineer. Roles during the study visit are defined in table 2.

Table 2 – Research team roles

Professional	Role in study	Description of responsibilities
Senior Anaesthetist	Medical cover	• Conduct medical screening<br>• Ensure participant safety throughout the study<br>• Inserting/removing radial arterial line<br>• Operating the hypoxicator equipment<br>• Taking ABG samples from arterial line

For peer review only

Researcher 1	Devices and Timestamping	• Ensure correct positioning of all involved devices<br>• Ensure data is being recorded from all monitors<br>• Timestamping of study activities and ABGs<br>• Troubleshoot any device-related issues throughout
Researcher 2	ABG processing	• Collect and process the ABG,<br>• Identify ABG report with correct activity (e.g. Tapping, Tablet, 95%)<br>• Discard the blood sample
Researcher 3	Participant Activities and Instructions	• Explain activities to participants<br>• Giving instructions and guide participants through movement phase activities<br>• Respiratory Rate manual count at ABG time points<br>• FiO_2 manual record at ABG time points in the Hypoxia phase
Researcher 4	Support/Backup	• Manually record the time ABGs are drawn<br>• Complement/assist any required activities<br>• Responsible for oversight and detection of any suboptimal activities/conditions
Engineer	Data monitoring	• Monitors procedures and real time data quality<br>• Double checks devices<br>• Ensures reliable data acquisition throughout

For peer review only

DATA COLLECTION AND MANAGEMENT

Devices

To ensure correct timestamping, Researcher 1 will verify all devices, tablets and laptops are connected to the same network. The time and date will be set to Greenwich Mean Time Zone (GMT) or British Summer Time (BST) as appropriate. The time will be verified to be within a tolerance of +/- 2 seconds and documented in the case report form (CRF):

1- AMDs

a. Vital Connect Inc. VitalPatch®; Single-use (120 hours), adhesive, wireless, waterproof patch that measures heart rate and respiratory rate via a single lead electrocardiogram (ECG/EKG). Other parameters include; 3 axis motion sensor and skin-temperature sensors.

b. Viatom Technology Co., Ltd. CheckMe™ O2; Wrist-worn wireless pulse oximeter, measuring pulse rate and percentage Oxygen saturation (SpO2) via transmittance photoplethysmography (PPG) using a ring-style sensor. Other parameters include a motion sensor.

c. Wavelet Health USA, LLC., Wavelet wristband; Wireless wrist-worn pulse oximeter using reflectance PPG to measure pulse rate and percentage Oxygen saturation (SpO2). Other parameters include 3-axis motion sensor and gyroscope.

d. Shenzhen Creative Industry Co. Ltd., AP-20; Wrist-worn wireless pulse oximeter, measuring pulse rate and percentage Oxygen (SpO2) via transmittance PPG using a finger-tip style sensor. Other parameters include estimation of respiratory rate using the airflow signal collected from a supplied nasal cannula (attached to an airflow sensor in the device); and 3-axis motion sensor.

e. Nonin Medical Inc., WristOX2 3150 BLE; Wrist-worn wireless pulse oximeter, measuring pulse rate and percentage of Oxygen (SpO2) via transmittance PPG with finger-tip style sensor.

2- **Clinical standard:**

a. Philips Monitor MX450 and extraction software (ixTrend 2.1):

3- **Gold standard:**

a. ABGs: The assigned person will manually record the GMT time when each ABG is taken. The ABG processing time will also be recorded as part of the automatic report. The ABGs will be analysed using a Radiometer ABL90 Flex blood gas analyser.

An electronic system, developed in-house, comprising a vital signs data collection application (app) running on Android tablets, and a web-application (administrated by the research group) will allow:

- the registration of the participant study number, centralised in the web-application, via the app;
- the collection of data from one patch (VitalPatch®) and one pulse-oximeter (AP-20, CheckMeO2, and WristOX2 3150), via Bluetooth Low-energy, and their storage into files in a tablet;
- the upload of the files from the tablet to the web-application server (via HTTPS) within 24 hours of the end of each session;
- the electronic recording of the timestamping of the activities in each phase of the study session (by Researcher 1), i.e.:
  - **Movement phase -** Normoxia / Sit to Stand / Tapping / Rubbing / Drinking / Turning / Tablet;
  - **Hypoxia phase -** 95% / 90% / 87% / 85% / 83% / 80% SpO2 levels.

A total of 3 tablets will be used to collect data from the VitalPatch®, CheckMe™ O2, WristOX2 3150 BLE, and the AP-20. Wavelet Health's electronic system will be used to collect data for the Wavelet device.

Collected data

The following data will be collected for each participant:

- Demographic data: including age, sex, height, weight, skin type (Fitzpatrick scale), baseline heart rate and SaO2 at start of test (using gold standard ABG measurements).
- For oxygen saturation, sampled at normoxia and each level of induced hypoxia:
  - **Gold standard reference:** ABGs (intermittent samples)
  - **Clinical standard reference:** Standard care pulse oximeter (continuous data)
  - **Devices under test**: Up to four pulse oximeters (continuous data)
- For pulse rate, sampled at normoxia and each level of induced hypoxia:
  - **Gold standard reference:** Arterial line trace (continuous data)
  - **Clinical standard reference:** Standard care pulse oximeter (continuous data)
  - **Devices under test**: Up to four pulse oximeters (continuous data)
- For heart rate, sampled at normoxia and each level of induced hypoxia:
  - **Gold standard reference and clinical standard reference**: Standard care 3-lead ECG (continuous data);
  - **Devices under test:** Chest patch (continuous data)
- For respiratory rate, sampled at normoxia and each level of induced hypoxia:
  - **Gold standard reference**: Capnography (continuous data)

For peer review only

- **Clinical standard reference**: Manual respiratory rate per minute counting (intermittent samples, done at the same time as the ABG sampling)
- **Devices under test:** Chest patch (continuous data)

Safety testing and calibration

Philips MX450, ABG machines, hypoxicator, all tablets and chargers were subjected to clinical safety testing by either the Department of Engineering Science , University of Oxford, or by the Clinical Engineering team at the Oxford University Hospitals NHS Foundation Trust (OUHFT). ABG analysers are maintained and calibrated by the Clinical Measurements team at the Oxford University Hospitals NHS Foundation Trust (OUHFT).

Data quality and completeness

Manually entered data (eg. ABG data, RR count, etc.) will be subject to a 10% data validation check. To ensure correct timestamping, all ABG collection times will be recorded both using the vHDU app and manually recorded time (hh:mm:ss). Each participant AMD, gold and clinical standard, and timestamp data will be plotted and audited visually up to one week after participation to assess data completeness. Each participant dataset will be deemed complete if there are test device data to answer either the primary or secondary objective, including both gold standard and clinical standard reference data.

ANALYSIS

For continuous data we will sample by two methods: (1) Simultaneous single data points, (corresponding to the time of ABG sampling where relevant) and;
(2) by selecting sampling windows of 5-30 seconds and comparing data for each device. Data points will be recorded across device timestamps to ensure accuracy of comparisons.

In accordance with the international standard of pulse oximeter equipment validation (ISO 80601-2-61:2019), the accuracy of the SpO2 measurement will be stated in terms of the root-mean-square (rms) difference between measured values (devices under test) (SpO2i) and reference values (gold standard arterial line and clinical standard) (SRi), as given by:

$$A_{rms} = \sqrt{\frac{\sum_{i=1}^{n}\left(SpO_{2i} - S_{Ri}\right)^2}{n}}$$

We will also compute the bias between gold standard, clinical standard and each device under test:

$$B = \frac{\sum_{i=1}^{n}\left(SpO_{2i} - S_{Ri}\right)}{n}$$

and the precision:

$$s_{res} = \sqrt{\frac{\sum_{i=1}^{n}\left(SpO_{2i} - SpO_{2fit,i}\right)^2}{(n-2)}}$$

where SpO2fit, is the value of the fitted curve corresponding to the ‘i’th reference value. Simple statistics about the difference among measurements (mean, standard deviation, percentiles, Bland-Altman plots) will be provided for the all the devices under analysis. Identical statistical methods will be applied to assess the agreement between the estimation of the (i) pulse rate from the pulse

For peer review only

oximeters, and of the (ii) heart rate and (iii) respiratory rate from the patch, and the corresponding reference measurements.

The performance of each pulse oximeter in detecting hypoxemia (at each level <=90%) will be assessed by reporting the optimal sensitivity and specificity pair, identified via Receiver-Operating Characteristic curves [7,12].

Descriptive statistics will be also computed per participant, per skin type and with and without movement artefacts.

Outcome analysis

We will analyse the outcomes on all the participants from whom we collected the data. The accuracy will be compared to the respective reference pulse oximeter. Where paired readings exist (device with clinical or gold standard) they will be included in the analysis. Device readings with no paired clinical or gold standard will be excluded from the analysis.

Primary outcome measure – Sensitivity and specificity for detecting hypoxia at each level <=90%): The analysis plan detailed above will provide data on correlation of each device with 'gold standard' arterial measurements, and their accuracy for the detection of hypoxemia: The output of this analysis will allow selection of the devices which correlate most closely to the 'gold standard' measurements, and provide the highest performance in the detection of hypoxemia.

Secondary outcome measure - Correlation of device outputs i.e. HR, RR, PR and SpO_2, with ECG derived HR, capnography derived RR, arterial blood pulse rate and pulse oximetry, respectively, during movement:

The analysis plan detailed above will provide data on correlation of each device with 'gold standard' arterial measurements during protocolised movement tests. The output of this analysis will allow selection of the devices which correlate most closely to the 'gold standard' measurements.

Safety reporting

A serious adverse event (SAE) occurring to a participant should be reported to the Research Ethics Committee (REC) that gave a favourable opinion of the study where in the opinion of the Chief Investigator the event was 'related' (resulted from administration of any of the research procedures) and 'unexpected' in relation to those procedures. Reports of related and unexpected SAEs should be submitted within 15 working days of the Chief Investigator becoming aware of the event, using the Health Regulatory Agency (HRA) report of serious adverse event form. The research team will also request participant's permission to contact the respective GP and report the SAE through the appropriate channels.

Informed Consent and participant withdrawals

Informed consent will be obtained by the lead researcher or a member of the research team (usually a research nurse/physiotherapist) at the start of the study visit (Appendix 3). All those obtaining consent will have received informed consent training as well as Good Clinical Practice training. Each participant has the right to withdraw from the study at any time, without giving a reason and without affecting their career or quality of their future care. If they wish to withdraw from the study, we will offer to destroy all gathered information. This will be possible up until the point where we de-identify participants' data.

Data Recording and pseudonymisation

All vital signs data will be collected as per each AMD (Appendix 2). Data derived from these devices will be limited to vital signs measurements and associated waveforms. These will be downloaded

For peer review only

from the device to a secure, password protected database. No personal identifiable information will be associated with these data. All data held will be associated with a de-identifiable participant number. Where data is uploaded initially to a cloud server (the Wavelet Health wristband), data will be subsequently downloaded by research staff. The download of data does not remove the de-identifiable data from the cloud storage. Access rights to the cloud data will be as per Cloud Privacy license. Participants will be explicitly advised as to the storage of de-identifiable vital signs data and that this data may be kept within the storage facility indefinitely. This will be made clear to participants prior to consent. Other AMD will record data directly to internal devices from which the data can be retrieved and deleted.

Linkage between pseudonym and identifying information will be held in one place, a password protected database on a networked secure server held by the University of Oxford. Access to this database will be limited to research nurses/allied health professionals only and will be destroyed at the end of the study, once all data has been verified. A spreadsheet will be maintained of de-identifiable participant baseline data, such as date of participation. No identifiable data will be held on this spreadsheet. This data will be entered and validated by the study researchers.

Any paper correspondence (such as CRFs and CFs) will be kept in the Kadoorie Centre in an established research area, behind two access-controlled doors and in locked filing cabinets. All documentation will be archived at the end of the project and retained for five years at the off-site secure archive facility (Re-Store) based at Upper Heyford.

Cloud Storage

As these are commercially available systems, de-identifiable data, with no personal identifiers may be transferred to Cloud storage. Where this is the case, this will be discussed with participants before connecting the equipment prior to consent. Data may remain on the storage system even when downloaded by the research team. Access to storage data is as per Cloud licensing agreement. Participants will be explicitly advised as to the storage of de-identifiable vital signs data and that this data may be kept within the storage facility indefinitely. Other AMD will record data directly to internal devices from which the data can be retrieved and deleted. For one device, the Wavelet Health wristband, de-identified data is transmitted to the device manufacturer's cloud-based system before we are able to access and download these data. There is no alternative to this method of transmission for this device.

Participant compensation

Participants will be reimbursed for travel costs incurred, plus appropriate payment in recognition for their time contribution to the study. They will receive vouchers to the monetary value of £20 for completing the pre-screening telephone interview, and then if willing and eligible to participate, £80 for the complete hypoxia exposure visit making a total of £100 for those who complete the study.

PATIENT AND PUBLIC INVOLVEMENT

This study is part of the vHDU project. During Phase 4 (commenced), we will develop a Patient and Public Involvement (PPI) group for ongoing support and feedback. We have attended a number of local Public Engagement Events, where members of the public showed genuine interest in the advances of wearable monitors and were engaged in our vision of a wireless hospital in the future.

References

1 McQuillan P, Pilkington S, Allan A, *et al.* Confidential inquiry into quality of care before admission to intensive care. *BMJ* 1998;**316**:1853–8. doi:10.1136/BMJ.316.7148.1853
2 Prgomet M, Cardona-Morrell M, Nicholson M, *et al.* Vital signs monitoring on general wards: clinical staff perceptions of current practices and the planned introduction of continuous

monitoring technology. *Int J Qual Heal Care* 2016;**28**:515–21. doi:10.1093/intqhc/mzw062
3 Jansen JO, Cuthbertson BH. Detecting critical illness outside the ICU: the role of track and trigger systems. *Curr Opin Crit Care* 2010;**16**:184–90. doi:10.1097/MCC.0b013e328338844e
4 Cardona-Morrell M, Prgomet M, Lake R, *et al.* Vital signs monitoring and nurse–patient interaction: A qualitative observational study of hospital practice. *Int J Nurs Stud* 2016;**56**:9–16. doi:10.1016/j.ijnurstu.2015.12.007
5 Weenk M, van Goor H, Frietman B, *et al.* Continuous Monitoring of Vital Signs Using Wearable Devices on the General Ward: Pilot Study. *JMIR mHealth uHealth* 2017;**5**:e91. doi:10.2196/mhealth.7208
6 Appelboom G, Camacho E, Abraham ME, *et al.* Smart wearable body sensors for patient self-assessment and monitoring. *Arch Public Heal* 2014;**72**:28. doi:10.1186/2049-3258-72-28
7 Louie A, Feiner JR, Bickler PE, *et al.* Four Types of Pulse Oximeters Accurately Detect Hypoxia during Low Perfusion and Motion. *Anesthesiology* 2018;**128**:520–30. doi:10.1097/ALN.0000000000002002
8 Chan A-W, Tetzlaff JM, Altman DG, *et al.* SPIRIT 2013 Statement: Defining Standard Protocol Items for Clinical Trials. *Ann Intern Med* 2013;**158**:200. doi:10.7326/0003-4819-158-3-201302050-00583
9 Hutter D, Kingdom J, Jaeggi E. Causes and mechanisms of intrauterine hypoxia and its impact on the fetal cardiovascular system: a review. *Int J Pediatr* 2010;**2010**:401323. doi:10.1155/2010/401323
10 Shenzhen Viatom Technology Co. L. O2 Vibe Wrist Pulse Oximeter Quick Start Guide. Shenzhen, P.R.China: 2016. https://fccid.io/2ADXK-1600/User-Manual/Users-Manual-3119531.pdf
11 Rowland MJ, Ezra M, Winkler A, *et al.* Calcium channel blockade with nimodipine reverses MRI evidence of cerebral oedema following acute hypoxia. *J Cereb Blood Flow Metab* 2017;:0271678X1772662. doi:10.1177/0271678X17726624
12 Kohyama T, Moriyama K, Kanai R, *et al.* Accuracy of Pulse Oximeters in Detecting Hypoxemia in Patients with Chronic Thromboembolic Pulmonary Hypertension. *PLoS One* 2015;**10**:e0126979. doi:10.1371/journal.pone.0126979

Authors' contributions

Authorship is determined in accordance with the ICMJE guidelines:
CA, SV, PP, LT and PW drafted the initial protocol. CA, SV, EK, JE, LY, OG, CR and MS will conduct the study procedures and data acquisition. AS and MH reviewed protocol and will provide medical cover in the study days. CA drafted the manuscript and all authors reviewed and approved it.
The funders have had no role in the study protocol design or the preparation of this manuscript, and will have no role in the collection, management, analysis and interpretation of the data, or the writing of the final report.

Competing interests statement.

PW and LT report significant grants from the National Institute of Health Research (NIHR), UK and the NIHR Biomedical Research Centre, Oxford, during the conduct of the study. PW and LT report modest grants and personal fees from Sensyne Health, outside the submitted work. PW and LT work part-time for Sensyne Health and hold shares in the company.

For peer review only

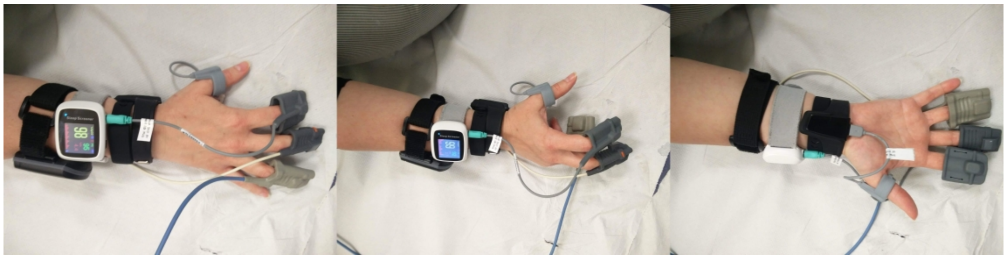

Devices placement example (dominant hand, combination 3)

381x95mm (300 x 300 DPI)

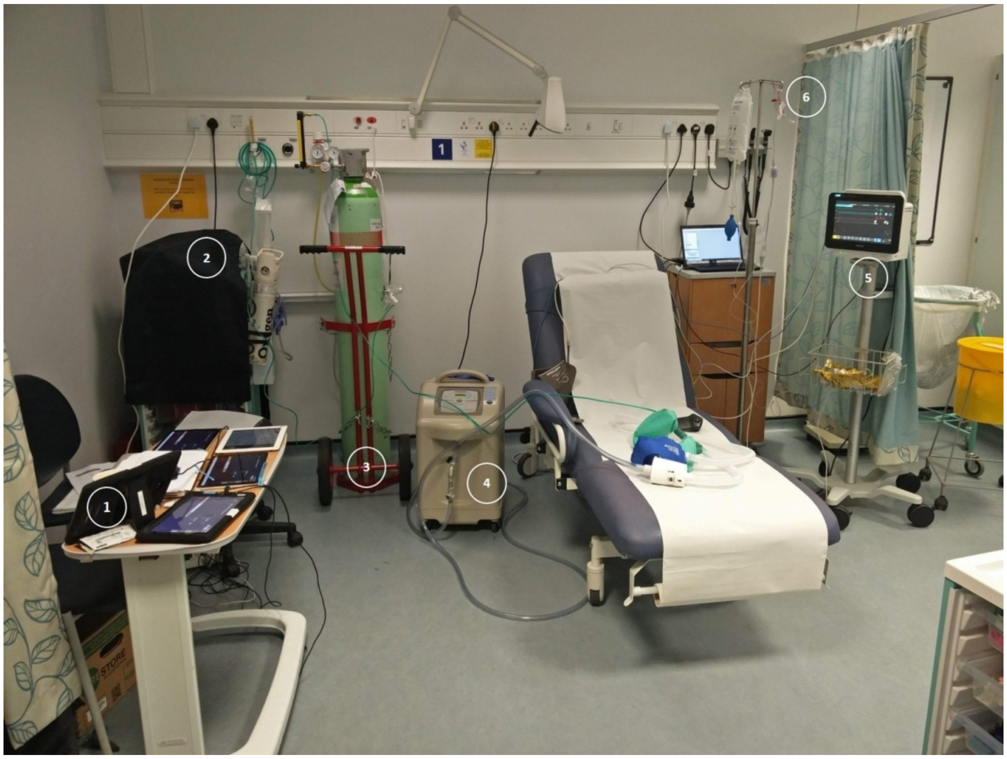

Hypoxia study day set up. Legend: 1- Tablets linked with AMD devices (4 Samsung Tab A, each linked with one AMD: AP-20, WristOX2 3150 BLE, CheckMe O2 and VitalPatch®. 1 IPad 4 connected to the Wavelet). 2- Resuscitation trolley and Oxygen. 3- 7% oxygen in nitrogen cylinder. 4- Hypoxicator apparatus. 5- Philips monitor (model MX450) connected to laptop (IX Trend software). 6- Drip stand with the arterial line pressure bag.

127x95mm (300 x 300 DPI)

Appendix 1: Functional Movement Testing – Tablet Protocol

Press home
Type in code _ _ _ _
Go to safari (compass icon)
Search 'Google'
Search 'Oxford weather forecast'
Select BBC weather
Scroll across hourly forecast and select 'see more weather for _______'
Scroll across again
Scroll down the page and select and play the BBC South Today weather video.
Press the full screen button in the right bottom corner of the screen. Watch the video then minimise the screen.
Go to the internet search bar and search 'google'
Search 'Amazon'
Select the Amazon website
Search 'Bicycle' and scroll to select a bicycle you like
Add to basket, then navigate back to the search page
Search 'Bicycle Helmet' and again scroll and select one.
Press the back button in the top left corner until back to the safari/google page
Type following sums:
100 x 70 / 45 =
80 + 6 + 95 + 43 + 51 + 15 =

For peer review only

Appendix 2: Wearable Ambulatory Monitors Summary

Monitors	Vital signs	Application	Monitoring method	Data storage	
**VitalPatch®**<br><br>**CE marked**	Respiratory rate (rpm), Heart rate (bpm)	Chest worn	1-lead ECG and accelerometer signals are used for the accurate estimation of heart rate and respiratory rate.	Data collected in real time when the device is synchronised via BLE with vHDU app, otherwise the data are recorded in the device's memory, and downloaded afterwards via the vHDU app.	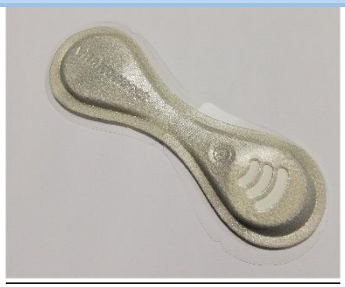
**CheckMe™ O2+**<br><br>**CE Marked**	SpO2 (%),<br>Pulse rate (bpm)	Wrist worn with thumb ring probe.	Transmittance PPG is used to estimate SpO2. The Infrared PPG is used to estimate pulse rate.	Data collected in real time when the device is synchronised via BLE with vHDU app.	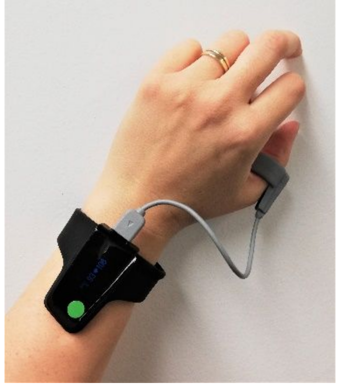

For peer review only

**AP-20®**<br><br>**CE Marked**	SpO2 (%),<br>Pulse rate (bpm),<br>Respiratory rate (rpm).	Wrist worn with finger-tip sensor. Includes Nasal flow sensor.	Transmittance PPG is used to estimate SpO2. The Infrared PPG is used to estimate pulse rate.<br><br>The airflow signal is used to determine the Respiratory Rate	Data collected in real time when the device is synchronised via BLE with vHDU app.<br><br>All data are also stored in the device memory, and downloaded afterwards via a the AP-20® software.	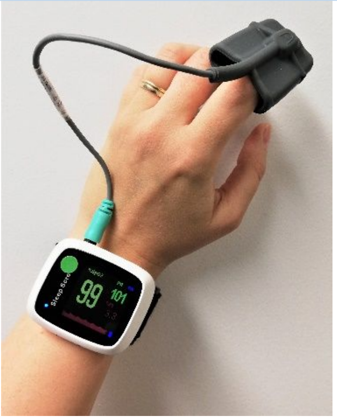
**Wavelet**<br><br>**No regulatory approval at the time of the study**	SpO2 (%),<br>Pulse rate (bpm)	Wrist worn	Reflectance PPG is used to estimate SpO2. The Infrared PPG is used to estimate pulse rate.<br><br>Accelerometer data is used to discard estimations perturbed by moment.	Wavelet onsite App is used to collect the data.<br><br>De-identifiable Data will be transferred to the Cloud storage but data may remain on the storage system even after being downloaded by the research team<br><br>Access to storage data is as per Cloud licensing agreement.	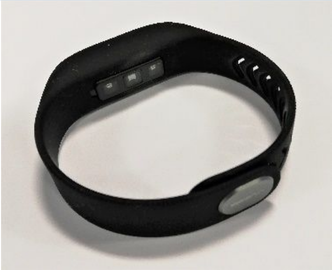
**WristOX2 3150 OEM BLE**<br><br><br>**FDA Approved**	SpO2 (%),<br>Pulse rate (bpm)	Wrist worn with finger-tip sensor.	Transmittance PPG is used to estimate SpO2. The Infrared PPG is used to estimate pulse rate.	Data collected in real time when the device is synchronised via BLE with vHDU app.	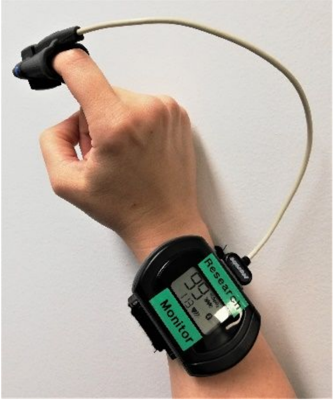

For peer review only

For peer review only

Appendix 3: Model Consent Form

Study Code: *Sub-Study code:* *Participant identification number:*

V	H	D	U	—	H	—			

CONSENT FORM

Virtual HDU: Hypoxia Study. Accuracy and validity testing of ambulatory monitoring system.

Name of Researcher: *If you agree, please initial box*

1. I confirm that I have read and understood the information sheet dated 17/JUN/2019 version 4.0 for the above study I have had the opportunity to consider the information, ask questions and have had these answered satisfactorily.	
2. I understand that my participation is voluntary and that I am free to withdraw at any time without giving any reason, without my medical care or legal rights being affected.	
3. I understand who will have access to personal data provided, how the data will be stored and what will happen to the data at the end of the project.	
4. I understand and consent for all required screening tests that will include medical history review, breathing tests, urine pregnancy test (female participants only) and blood sample tests.	
5. I agree to physiological vital sign monitoring with the use of ambulatory monitoring device(s).	
6. I agree to cannulation (insertion of tube in your radial artery) and hypoxic exposure (controlled reduction of oxygen levels) for the duration of the testing phase of the study and understand these procedures and potential (although very rare) complications.	
7. I agree to donate up to 15(teaspoon-sized) blood samples. I consider these samples a gift to the University of Oxford and I understand I will not gain any direct personal or financial benefit from them. I also understand these will be discarded and not retained by the research team after the study visit.	
8. I understand that in some cases the monitoring systems being used require initial upload of vital signs data to their proprietary Cloud storage facility that might be abroad, from which these data are then downloaded. I understand in this case this will be discussed with me beforehand and no identifiable data will be included in this upload.	
9. I understand how to raise a concern and make a complaint.	
**10. I agree to take part in this study.**	

______________________ ________________ __________________________

Name of Participant *Date* *Signature*

______________________ ________________ __________________________

Name of Person taking Consent *Date* *Signature*

**1 copy for participant; 1 copy for researcher site file; 1 (original) to be kept in medical notes* (if participant is a patient).

For peer review only

SPIRIT
STANDARD PROTOCOL ITEMS: RECOMMENDATIONS FOR INTERVENTIONAL TRIALS

SPIRIT 2013 Checklist: Recommended items to address in a clinical trial protocol and related documents*

Section/item	Item No	Description	Addressed on page number
**Administrative information**			
Title	1	Descriptive title identifying the study design, population, interventions, and, if applicable, trial acronym	1
Trial registration	2a	Trial identifier and registry name. If not yet registered, name of intended registry	2
	2b	All items from the World Health Organization Trial Registration Data Set	Throughout
Protocol version	3	Date and version identifier	2
Funding	4	Sources and types of financial, material, and other support	2
Roles and responsibilities	5a	Names, affiliations, and roles of protocol contributors	1, 12
	5b	Name and contact information for the trial sponsor	2
	5c	Role of study sponsor and funders, if any, in study design; collection, management, analysis, and interpretation of data; writing of the report; and the decision to submit the report for publication, including whether they will have ultimate authority over any of these activities	12
	5d	Composition, roles, and responsibilities of the coordinating centre, steering committee, endpoint adjudication committee, data management team, and other individuals or groups overseeing the trial, if applicable (see Item 21a for data monitoring committee)	N/A

For peer review only

**Introduction**			
Background and rationale	6a	Description of research question and justification for undertaking the trial, including summary of relevant studies (published and unpublished) examining benefits and harms for each intervention	3
	6b	Explanation for choice of comparators	3
Objectives	7	Specific objectives or hypotheses	3
Trial design	8	Description of trial design including type of trial (eg, parallel group, crossover, factorial, single group), allocation ratio, and framework (eg, superiority, equivalence, noninferiority, exploratory)	3,4
**Methods: Participants, interventions, and outcomes**			
Study setting	9	Description of study settings (eg, community clinic, academic hospital) and list of countries where data will be collected. Reference to where list of study sites can be obtained	7
Eligibility criteria	10	Inclusion and exclusion criteria for participants. If applicable, eligibility criteria for study centres and individuals who will perform the interventions (eg, surgeons, psychotherapists)	4
Interventions	11a	Interventions for each group with sufficient detail to allow replication, including how and when they will be administered	5-7
	11b	Criteria for discontinuing or modifying allocated interventions for a given trial participant (eg, drug dose change in response to harms, participant request, or improving/worsening disease)	5
	11c	Strategies to improve adherence to intervention protocols, and any procedures for monitoring adherence (eg, drug tablet return, laboratory tests)	5-7
	11d	Relevant concomitant care and interventions that are permitted or prohibited during the trial	N/A
Outcomes	12	Primary, secondary, and other outcomes, including the specific measurement variable (eg, systolic blood pressure), analysis metric (eg, change from baseline, final value, time to event), method of aggregation (eg, median, proportion), and time point for each outcome. Explanation of the clinical relevance of chosen efficacy and harm outcomes is strongly recommended	5-7
Participant timeline	13	Time schedule of enrolment, interventions (including any run-ins and washouts), assessments, and visits for participants. A schematic diagram is highly recommended (see Figure)	6,7

For peer review only - http://bmjopen.bmj.com/site/about/guidelines.xhtml

Sample size	14	Estimated number of participants needed to achieve study objectives and how it was determined, including clinical and statistical assumptions supporting any sample size calculations	4
Recruitment	15	Strategies for achieving adequate participant enrolment to reach target sample size	4

Methods: Assignment of interventions (for controlled trials)

Allocation:			
Sequence generation	16a	Method of generating the allocation sequence (eg, computer-generated random numbers), and list of any factors for stratification. To reduce predictability of a random sequence, details of any planned restriction (eg, blocking) should be provided in a separate document that is unavailable to those who enrol participants or assign interventions	N/A
Allocation concealment mechanism	16b	Mechanism of implementing the allocation sequence (eg, central telephone; sequentially numbered, opaque, sealed envelopes), describing any steps to conceal the sequence until interventions are assigned	N/A
Implementation	16c	Who will generate the allocation sequence, who will enrol participants, and who will assign participants to interventions	N/A
Blinding (masking)	17a	Who will be blinded after assignment to interventions (eg, trial participants, care providers, outcome assessors, data analysts), and how	N/A
	17b	If blinded, circumstances under which unblinding is permissible, and procedure for revealing a participant's allocated intervention during the trial	N/A

Methods: Data collection, management, and analysis

Data collection methods	18a	Plans for assessment and collection of outcome, baseline, and other trial data, including any related processes to promote data quality (eg, duplicate measurements, training of assessors) and a description of study instruments (eg, questionnaires, laboratory tests) along with their reliability and validity, if known. Reference to where data collection forms can be found, if not in the protocol	8-9
	18b	Plans to promote participant retention and complete follow-up, including list of any outcome data to be collected for participants who discontinue or deviate from intervention protocols	N/A

Data management	19	Plans for data entry, coding, security, and storage, including any related processes to promote data quality (eg, double data entry; range checks for data values). Reference to where details of data management procedures can be found, if not in the protocol	10-12
Statistical methods	20a	Statistical methods for analysing primary and secondary outcomes. Reference to where other details of the statistical analysis plan can be found, if not in the protocol	10,11
	20b	Methods for any additional analyses (eg, subgroup and adjusted analyses)	N/A
	20c	Definition of analysis population relating to protocol non-adherence (eg, as randomised analysis), and any statistical methods to handle missing data (eg, multiple imputation)	10
**Methods: Monitoring**			
Data monitoring	21a	Composition of data monitoring committee (DMC); summary of its role and reporting structure; statement of whether it is independent from the sponsor and competing interests; and reference to where further details about its charter can be found, if not in the protocol. Alternatively, an explanation of why a DMC is not needed	N/A
	21b	Description of any interim analyses and stopping guidelines, including who will have access to these interim results and make the final decision to terminate the trial	N/A
Harms	22	Plans for collecting, assessing, reporting, and managing solicited and spontaneously reported adverse events and other unintended effects of trial interventions or trial conduct	11
Auditing	23	Frequency and procedures for auditing trial conduct, if any, and whether the process will be independent from investigators and the sponsor	10
**Ethics and dissemination**			
Research ethics approval	24	Plans for seeking research ethics committee/institutional review board (REC/IRB) approval	2
Protocol amendments	25	Plans for communicating important protocol modifications (eg, changes to eligibility criteria, outcomes, analyses) to relevant parties (eg, investigators, REC/IRBs, trial participants, trial registries, journals, regulators)	2

For peer review only - http://bmjopen.bmj.com/site/about/guidelines.xhtml

Consent or assent	26a	Who will obtain informed consent or assent from potential trial participants or authorised surrogates, and how (see Item 32)	12
	26b	Additional consent provisions for collection and use of participant data and biological specimens in ancillary studies, if applicable	N/A
Confidentiality	27	How personal information about potential and enrolled participants will be collected, shared, and maintained in order to protect confidentiality before, during, and after the trial	12
Declaration of interests	28	Financial and other competing interests for principal investigators for the overall trial and each study site	13
Access to data	29	Statement of who will have access to the final trial dataset, and disclosure of contractual agreements that limit such access for investigators	12
Ancillary and post-trial care	30	Provisions, if any, for ancillary and post-trial care, and for compensation to those who suffer harm from trial participation	12
Dissemination policy	31a	Plans for investigators and sponsor to communicate trial results to participants, healthcare professionals, the public, and other relevant groups (eg, via publication, reporting in results databases, or other data sharing arrangements), including any publication restrictions	2
	31b	Authorship eligibility guidelines and any intended use of professional writers	13,14
	31c	Plans, if any, for granting public access to the full protocol, participant-level dataset, and statistical code	2
**Appendices**			
Informed consent materials	32	Model consent form and other related documentation given to participants and authorised surrogates	
Biological specimens	33	Plans for collection, laboratory evaluation, and storage of biological specimens for genetic or molecular analysis in the current trial and for future use in ancillary studies, if applicable	

*It is strongly recommended that this checklist be read in conjunction with the SPIRIT 2013 Explanation & Elaboration for important clarification on the items. Amendments to the protocol should be tracked and dated. The SPIRIT checklist is copyrighted by the SPIRIT Group under the Creative Commons "Attribution-NonCommercial-NoDerivs 3.0 Unported" license.

Protocol for a prospective, controlled, cross-sectional, diagnostic accuracy study to evaluate the specificity and sensitivity of ambulatory monitoring systems in the prompt detection of hypoxia and during movement.

Journal:	*BMJ Open*
Manuscript ID	bmjopen-2019-034404.R1
Article Type:	Protocol
Date Submitted by the Author:	12-Dec-2019

Complete List of Authors:	Areia, Carlos; University of Oxford, Nuffield Department of Clinical Neurosciences<br>Vollam, Sarah; University of Oxford, Nuffield Department of Clinical Neurosciences<br>Piper, Philippa; Oxford University Hospitals NHS Foundation Trust, Adult Intensive Care Unit<br>King, Elizabeth; Oxford University Hospitals NHS Foundation Trust, Adult Intensive Care Unit<br>Ede, Jody; University of Oxford, Nuffield Department of Clinical Neurosciences<br>Young, Louise; University of Oxford, Nuffield Department of Clinical Neurosciences<br>Santos, Mauro; University of Oxford, Institute of Biomedical Engineering, Department of Engineering Science<br>Pimentel, Marco; University of Oxford, Institute of Biomedical Engineering, Department of Engineering Science<br>Roman, Cristian; University of Oxford, Institute of Biomedical Engineering, Department of Engineering Science<br>Harford, Mirae; University of Oxford, Nuffield Department of Clinical Neurosciences<br>Shah, Akshay; University of Oxford, Radcliffe Department of Medicine<br>Gustafson, Owen; Oxford University Hospitals NHS Foundation Trust, Adult Intensive Care Unit<br>Rowland, Matthew; University of Oxford, Nuffield Department of Clinical Neurosciences<br>Tarassenko, Lionel; University of Oxford, Institute of Biomedical Engineering, Department of Engineering Science<br>Watkinson, Peter; University of Oxford, Nuffield Department of Clinical Neurosciences

**Primary Subject Heading**:	Diagnostics
Secondary Subject Heading:	Anaesthesia, Health informatics, Intensive care, Research methods
Keywords:	Adult intensive < critical care < ANAESTHETICS, INTENSIVE & CRITICAL CARE, Health informatics < BIOTECHNOLOGY & BIOINFORMATICS, BIOTECHNOLOGY & BIOINFORMATICS

For peer review only - http://bmjopen.bmj.com/site/about/guidelines.xhtml

SCHOLARONE™
Manuscripts

Protocol for a prospective, controlled, cross-sectional, diagnostic accuracy study to evaluate the specificity and sensitivity of ambulatory monitoring systems in the prompt detection of hypoxia and during movement.

Carlos Areia[1], Sarah Vollam[1], Philippa Piper[2], Elizabeth King[2], Jody Ede[1], Louise Young[1], Mauro Santos[3], Marco Pimentel[3], Cristian Roman[3], Mirae Harford[1], Akshay Shah[4], Owen Gustafson[2], Matthew Rowland[1], Lionel Tarassenko[3], Peter Watkinson[1]

1 – Critical Care Research Group, Nuffield Department of Clinical Neurosciences, University of Oxford, Oxford, United Kingdom
2 – Adult Intensive Care Unit, Oxford University Hospitals NHS Foundation Trust, Oxford, United Kingdom
3 – Institute of Biomedical Engineering, Department of Engineering Science, University of Oxford, Oxford, United Kingdom
4 – Radcliffe Department of Medicine, University of Oxford, Oxford, United Kingdom

Corresponding author:
Carlos Areia,
Critical Care Research Group, NIHR Oxford Biomedical Research Centre
Kadoorie Centre, Level 3 John Radcliffe Hospital
Oxford, United Kingdom.
OX3 9DU

carlos.morgadoareia@ndcn.ox.ac.uk

For peer review only

ABSTRACT

Introduction: Automated continuous ambulatory monitoring may provide an alternative to intermittent manual vital signs monitoring. This has the potential to improve frequency of measurements, timely escalation of care and patient safety. However, a major barrier to the implementation of these wearable devices in the ward environment is their uncertain reliability, efficiency and data fidelity. The purpose of this study is to test performance of selected devices in a simulated clinical setting including during movement and low levels of peripheral oxygen saturation.

Methods and Analysis: This is a single centre, prospective, controlled, cross-sectional, diagnostic accuracy study to determine the specificity and sensitivity of currently available ambulatory vital signs monitoring equipment in the detection of hypoxia and the effect of movement on data acquisition. We will recruit up to 45 healthy volunteers that will attend a single study visit; starting with a movement phase and followed by the hypoxia exposure phase where we will gradually decrease saturation levels down to 80%. We will simultaneously test one chest patch, one wrist worn only and three wrist worn with finger probe devices against 'clinical standard 'and 'gold standard' references. We will measure peripheral oxygen saturations, pulse rate, heart rate and respiratory rate continuously and arterial blood gases intermittently throughout the study.

Ethics and Dissemination: This study has received ethical approval by the East of Scotland Research Ethics Service REC 2 (19/ES/0008). The results will be broadly distributed through conference presentations and peer-reviewed publications.

Keywords: Hypoxia, ambulatory monitoring, vital signs, wearable devices

ARTICLE SUMMARY

Strengths and Limitations of this study

- Controlled hypoxia exposure in a standardised environment for all participants
- Outcome comparison to both clinical and gold standards
- Largest study in healthy volunteers
- Once specificity and sensitivity have been established in healthy volunteers; devices will be tested in the target hospital population

Word count: 4057

Protocol Version: 1.0
Protocol Date: 07 Aug 2019
Public Title: Testing the effects of low levels of blood oxygen and movement in the accuracy of wearable vital signs monitors
Trial Registration number: ISRCTN61535692 registered on 10/06/2019
Sponsorship: University of Oxford. Clinical Trials & Research Governance (ctrg@admin.ox.ac.uk)
Funding: This study/project is funded by the NIHR Biomedical Research Centre, Oxford. PW and LT are supported by the NIHR Biomedical Research Centre, Oxford.
Disclaimer: The views expressed are those of the authors and not necessarily those of the NHS, the NIHR or the Department of Health.
Recruitment start date: 18 Jun 2019
Status: Recruiting

For peer review only

INTRODUCTION

Failure to recognise and act on physiological indicators of worsening acute illness in hospital wards is a prevalent problem recognised over twenty years ago [1]. Current practice involves the use of early warning scoring systems, which monitor standard vital signs. These include intermittent measurements of pulse rate, respiratory rate, blood pressure, oxygen saturations and temperature. The frequency of vital signs measurements is usually guided by the clinical condition of the patient.

Intermittent measurement of these vital signs can be time consuming for healthcare professionals [2] and therefore the desired frequency of observations is often not achieved [3]. Infrequent measurement of vital signs may also miss clinical deteriorations between these measurements [4]. Thus, more sustainable, accurate and less time-consuming monitoring methods would be highly desirable.

Wearable ambulatory monitors (AM) may provide an alternative to intermittent vital signs monitoring by enabling the continuous monitoring of vital signs parameters. In addition to reducing the burden of intermittent measurement of vital signs on staff, continuous monitoring has the potential to facilitate earlier detection of deranged physiological parameters [5]. A major barrier to the clinical implementation of these wearable devices is their uncertain reliability, efficiency and data fidelity [6]. In particular, the effect of motion on its accuracy is under-investigated. Recent work by Louie et al (2018) tested four non-ambulatory pulse oximeters and found that motion impaired performance throughout a clinically relevant range of measurements. Less accuracy was reported at lower arterial oxygen saturations, which is undesirable in clinical practice [7].

This study is part of the Virtual High Dependency Unit (vHDU) project, a collaboration between the Institute of Biomedical Engineering and clinicians from the Nuffield Department of clinical Neurosciences at the University of Oxford. This is a phased project aiming to refine and integrate ambulatory monitoring systems for use in clinical practice. Previous phases have tested device wearability and in situ testing on hospital wards. The purpose of this study is to test the performance of selected devices in a simulated clinical setting which will involve participant movement and inducing low peripheral oxygen saturations.

METHODS

This protocol follows the “Standard Protocol Items: Recommendations for Interventional Trials” (SPIRIT) reporting guidelines [8]. We registered this protocol in a public database: ISRCTN61535692.

Study objectives

Primary objective: To determine the specificity and sensitivity of currently available ambulatory vital signs monitoring equipment for the detection of hypoxia.

Secondary objective: To determine the effect of movement on data acquisition by currently available ambulatory vital signs monitoring equipment.

These objectives will be assessed by comparing continuous peripheral oxygen saturations, pulse rate, heart rate and respiratory rate data from each ambulatory monitor with arterial blood oxygen saturation measured through arterial blood sampling, pulse rate derived from the arterial blood signal, heart rate derived from standard care 3-lead ECG, and respiratory rate derived from capnography and manual counting.

Study Design

For peer review only

Prospective, observational cross-sectional cohort study. Vital signs parameters from study devices will be compared with 'gold standard' and 'clinical standard' measurements.

Sample size

Our sample size calculation is based on the ISO 80601-2-61:2019 guideline for pulse-oximetry equipment accuracy testing. This requires at least 200 data points balanced across each decadal range (70 - 80%, 80 - 90%, 90 - 100%) of the SaO_2 range 70 – 100%, from at least 10 subjects. Approximately 30 full data sets will be required, to yield sufficient data points for the primary and secondary outcomes; therefore up to 45 healthy adult subjects who meet the inclusion criteria will participate in the study. For the broadest application to the largest group of participants, the subjects should vary in their physical characteristics to the greatest extent possible.

Recruitment

Up to 45 healthy volunteers will be recruited, with adverts placed in appropriate target locations such as college common spaces and university buildings. The adverts will contain a description of the study and the number and email contacts of one of the members of the research team.

Inclusion criteria for the study are: willing and able to give informed consent for participation in the study; men and women aged 18 or over; and in generally good health.

Exclusion criteria are: allergies to adhesive dressings (such as bio-occlusive dressings or micropore) or local anaesthetic (e.g. lidocaine); intra-cardiac device (e.g. Permanent pacemaker) or previous wrist arterial line; epilepsy; angina, congenital heart disease or history of severe cardio-pulmonary disease; history of anaemia (reported in the pre-screening telephone call), haemoglobinopathy or haemoglobin below 100 g/l on first test; resting hypoxaemia (SpO_2 <94%) or significant cardiopulmonary disease rendering exposure to alveolar hypoxia unsafe, as determined by the research physician; pregnancy or breast feeding; clotting disorders and use of antiplatelet or anticoagulant medication (such as aspirin); and claustrophobia precluding spell in the hypoxic exposure.

Study procedures

Initial Contact

Healthy volunteers will contact the research team via telephone/email to express their interest in the study. The research team will provide further information including the Participant Information Sheet (PIS). If volunteers wish to proceed, a telephone appointment will be arranged to complete a brief pre-screening assessment with a research nurse/physiotherapist (supported by a senior anaesthetist).

Pre-screening assessment

During the pre-screening telephone appointment, the study will be discussed further and general screening questions will be asked, to confirm eligibility. Questions will be encouraged to ensure the potential participant understands the study. If the potential participant agrees to take part, an appointment for the hypoxia exposure visit will be agreed.

Study visit

Screening Assessment

The screening assessment will be completed by an appropriately qualified, medically trained member of the research team, who will confirm eligibility for the hypoxic exposure phase. This will include a urinary pregnancy test for all female participants of child bearing potential. Pregnancy is an exclusion criteria for the study as the effects of hypoxia on pregnancy are unknown [9].

For peer review only

Arterial Line Insertion
After confirmation of eligibility, an arterial line will be inserted into the non-dominant radial artery of each participant on the day of the study visit under local anaesthesia.

Initial blood gas sampling
The first arterial blood gas (ABG) measurement will be assessed by the anaesthetist to confirm haemoglobin concentration ≥ 100 g/l. If the haemoglobin is below this level, the participant will be withdrawn from the study, the arterial line removed and the participant advised to discuss this finding with their general practitioner (as stated in the PIS).

Placement of devices
Participants will wear several ambulatory monitoring devices (AMD) which may include a chest worn patch (VitalPatch®), a purely wrist worn device (Wavelet) and up to 3 wrist worn devices with finger probe(CheckMe™ O2+, AP-20 and WristOX2 3150 BLE). The AMD detect various combinations of pulse oximetry, pulse rate, heart rate and respiratory rate (please refer to Appendix 1 for device details). Participants will be asked to wear a maximum of 5 study devices during the hypoxia exposure visit (Figure 1).

Finger probe position randomisation
Up to three study device finger probes will be worn, in addition to the 'clinical standard' reference bedside monitor finger probe. The CheckMeTM O2+ will always be worn on the thumb as per manufacturer recommendation [10]. To ensure parity of testing, the position of the other three finger probes on the 2nd, 3rd and 4th fingers will be randomised (using https://www.random.org/) per study visit day, ensuring an even distribution of placement, as per Table 1).

Table 1 – Device combinations

**Devices placement**	**Combination 1 (n=10)**	**Combination 2 (n=10)**	**Combination 3 (n=10)**
**Chest**	VitalPatch®	VitalPatch®	VitalPatch®
**Wrist**	Wavelet	Wavelet	Wavelet
**1st (thumb)**	CheckMe™ O2+	CheckMe™ O2+	CheckMe™ O2+
**2nd (index finger)**	Philips monitor (MX450)	WristOX2 3150 BLE	AP-20
**3rd (middle finger)**	AP-20	Philips monitor (MX450)	WristOX2 3150 BLE
**4th (ring finger)**	WristOX2 3150 BLE	AP-20	Philips monitor (MX450)

Stage 1 - Movement phase
During the movement phase participants will be seated in a chair and asked to complete a series of consecutive standardised movements, as detailed in Table 2. An ABG and manual (counted) respiratory rate will be taken at the end of each movement.

Table 2 - Standardised movements to be tested

**Movement**	**Task**
Standing from chair using arms to push up/sit down	20x repetitions

Tapping	Volunteer to tap a surface with all test side fingers simultaneously at the speed of a metronome set at 100 bpm for 2 minutes
Rubbing	Volunteer to complete a sideways rubbing movement with all test side fingers simultaneously at the speed of a metronome set at 100 bpm for 2 minutes
Drinking from plastic cup	20 x lift/drink/put down
Turning page	50x page turns
Using tablet	As per protocoled instructions (Appendix 2)

Stage 2 - Hypoxia Exposure phase
Participants will move to a bed and lie comfortably in a semi-recumbent, supine position (Figure 2). A tight-fitting silicone facemask will be placed and connected to a hypoxicator unit (Everest Summit Hypoxic Generator, www.altitudecentre.com). If required, additional 7% oxygen in nitrogen from a cylinder will be entrained into the hypoxicator circuit to ensure tight control of fraction of inspired oxygen (FiO_2) provided to the participant [11]. Inhaled FiO_2 will be monitored by an in-line gas analyser and end-tidal carbon dioxide ($etCO_2$) will be also recorded via capnography using the Philips monitor MX450 (www.philips.co.uk).

During the hypoxia exposure phase, oxygen saturations from the 'clinical standard' Philips monitor will guide the titration of the hypoxicator. 7% oxygen in nitrogen will be used to further lower FiO_2 if required. An ABG will be sampled when the participant reaches and remains stable at each pre-specified target peripheral oxygen saturation level (95%, 90%, 87%, 85%, 83%, 80%). We specified these saturations to allow assessment for our use case of prompt detection of hypoxia in normal adult patients in a ward environment, including multiple assessments within the 83-95% range, and one assessment at the top end of the 70-80% range, considered severe hypoxia.

Blood sampling
Up to 15 ABG samples will be taken. Samples will be discarded at the end of the laboratory session into clinical waste and no blood will be retained by the study.

At the end of the study visit the arterial line will be removed and firm pressure applied to the site until haemostasis is achieved. A sterile dressing will be applied and advice given to the participant on action to take if any bleeding occurs.

Facilities and Research Staff
Facilities
All study visits will occur in the Cardiovascular Clinical Research Facility, Level 1 Oxford Heart Centre, John Radcliffe Hospital, Headley Way, Headington, Oxford, OX3 9DU.

Roles and Responsibilities
Each study visit day will be staffed by one senior anaesthetist, four clinical researchers and one engineer. Roles during the study visit are defined in table 3.

Table 3 – Research team roles

**Professional**	**Role in study**	**Description of responsibilities**
Senior Anaesthetist	Medical cover	• Conduct medical screening

For peer review only

		• Ensure participant safety throughout the study<br>• Inserting/removing radial arterial line<br>• Operating the hypoxicator equipment<br>• Taking ABG samples from arterial line
Researcher 1	Devices and Timestamping	• Ensure correct positioning of all involved devices<br>• Ensure data is being recorded from all monitors<br>• Timestamping of study activities and ABGs<br>• Troubleshoot any device-related issues throughout
Researcher 2	ABG processing	• Collect and process the ABG,<br>• Identify ABG report with correct activity (e.g. Tapping, Tablet, 95%)<br>• Discard the blood sample
Researcher 3	Participant Activities and Instructions	• Explain activities to participants<br>• Giving instructions and guide participants through movement phase activities<br>• Respiratory Rate manual count at ABG time points<br>• FiO_2 manual record at ABG time points in the Hypoxia phase
Researcher 4	Support/Backup	• Manually record the time ABGs are drawn<br>• Complement/assist any required activities<br>• Responsible for oversight and detection of any suboptimal activities/conditions
Engineer	Data monitoring	• Monitors procedures and real time data quality<br>• Double checks devices<br>• Ensures reliable data acquisition throughout

DATA COLLECTION AND MANAGEMENT

Devices

To ensure correct timestamping, Researcher 1 will verify all devices, tablets and laptops are connected to the same network. The time and date will be set to Greenwich Mean Time Zone (GMT) or British Summer Time (BST) as appropriate. The time will be verified to be within a tolerance of +/- 2 seconds and documented in the case report form (CRF):

1- AMDs

a. Vital Connect Inc. VitalPatch®; Single-use (120 hours), adhesive, wireless, waterproof patch that measures heart rate and respiratory rate via a single lead electrocardiogram (ECG/EKG). Other parameters include; 3 axis motion sensor and skin-temperature sensors.

b. Viatom Technology Co., Ltd. CheckMe™ O2; Wrist-worn wireless pulse oximeter, measuring pulse rate and percentage Oxygen saturation (SpO2) via transmittance photoplethysmography (PPG) using a ring-style sensor. Other parameters include a motion sensor.

c. Wavelet Health USA, LLC., Wavelet wristband; Wireless wrist-worn pulse oximeter using reflectance PPG to measure pulse rate and percentage Oxygen saturation (SpO2). Other parameters include 3-axis motion sensor and gyroscope.

d. Shenzhen Creative Industry Co. Ltd., AP-20; Wrist-worn wireless pulse oximeter, measuring pulse rate and percentage Oxygen (SpO2) via transmittance PPG using a finger-tip style sensor. Other parameters include estimation of respiratory rate using

For peer review only

the airflow signal collected from a supplied nasal cannula (attached to an airflow sensor in the device); and 3-axis motion sensor.

e. Nonin Medical Inc., WristOX2 3150 BLE; Wrist-worn wireless pulse oximeter, measuring pulse rate and percentage of Oxygen (SpO2) via transmittance PPG with finger-tip style sensor.

2- **Clinical standard:**
   a. Philips Monitor MX450 and extraction software (ixTrend 2.1):

3- **Gold standard:**
   a. ABGs: The assigned person will manually record the GMT time when each ABG is taken. The ABG processing time will also be recorded as part of the automatic report. The ABGs will be analysed using a Radiometer ABL90 Flex blood gas analyser.

An electronic system, developed in-house, comprising a vital signs data collection application (app) running on Android tablets, and a web-application (administrated by the research group) will allow:

- the registration of the participant study number, centralised in the web-application, via the app;
- the collection of data from one patch (VitalPatch®) and one pulse-oximeter (AP-20, CheckMeO2, and WristOX2 3150), via Bluetooth Low-energy, and their storage into files in a tablet;
- the upload of the files from the tablet to the web-application server (via HTTPS) within 24 hours of the end of each session;
- the electronic recording of the timestamping of the activities in each phase of the study session (by Researcher 1), i.e.:
  - **Movement phase -** Normoxia / Sit to Stand / Tapping / Rubbing / Drinking / Turning / Tablet;
  - **Hypoxia phase -** 95% / 90% / 87% / 85% / 83% / 80% SpO2 levels.

A total of 3 tablets will be used to collect data from the VitalPatch®, CheckMe™ O2, WristOX2 3150 BLE, and the AP-20. Wavelet Health's electronic system will be used to collect data for the Wavelet device.

Collected data

The following data will be collected for each participant:

- Demographic data: including age, sex, height, weight, skin type (Fitzpatrick scale), baseline heart rate and SaO2 at start of test (using gold standard ABG measurements).
- For oxygen saturation, sampled at normoxia and each level of induced hypoxia:
  - **Gold standard reference:** ABGs (intermittent samples)
  - **Clinical standard reference:** Standard care pulse oximeter (continuous data)
  - **Devices under test**: Up to four pulse oximeters (continuous data)
- For pulse rate, sampled at normoxia and each level of induced hypoxia:
  - **Gold standard reference:** Arterial line trace (continuous data)
  - **Clinical standard reference:** Standard care pulse oximeter (continuous data)
  - **Devices under test**: Up to four pulse oximeters (continuous data)
- For heart rate, sampled at normoxia and each level of induced hypoxia:
  - **Gold standard reference and clinical standard reference**: Standard care 3-lead ECG (continuous data);
  - **Devices under test:** Chest patch (continuous data)
- For respiratory rate, sampled at normoxia and each level of induced hypoxia:

For peer review only

- **Gold standard reference**: Capnography (continuous data)
- **Clinical standard reference**: Manual respiratory rate per minute counting (intermittent samples, done at the same time as the ABG sampling)
- **Devices under test:** Chest patch (continuous data)

Safety testing and calibration
Philips MX450, ABG machines, hypoxicator, all tablets and chargers were subjected to clinical safety testing by either the Department of Engineering Science , University of Oxford, or by the Clinical Engineering team at the Oxford University Hospitals NHS Foundation Trust (OUHFT). ABG analysers are maintained and calibrated by the Clinical Measurements team at the Oxford University Hospitals NHS Foundation Trust (OUHFT).

Data quality and completeness
Manually entered data (eg. ABG data, RR count, etc.) will be subject to a 10% data validation check. To ensure correct timestamping, all ABG collection times will be recorded both using the vHDU app and manually recorded time (hh:mm:ss). Each participant AMD, gold and clinical standard, and timestamp data will be plotted and audited visually up to one week after participation to assess data completeness. Each participant dataset will be deemed complete if there are test device data to answer either the primary or secondary objective, including both gold standard and clinical standard reference data.

ANALYSIS
For continuous data we will sample by two methods: (1) Simultaneous single data points, (corresponding to the time of ABG sampling where relevant) and;
(2) by selecting sampling windows of 5-30 seconds and comparing data for each device. Data points will be recorded across device timestamps to ensure accuracy of comparisons.

In accordance with the international standard of pulse oximeter equipment validation (ISO 80601-2-61:2019), the accuracy of the SpO2 measurement will be stated in terms of the root-mean-square (rms) difference between measured values (devices under test) (SpO2i) and reference values (gold standard arterial line and clinical standard) (SRi), as given by:

$$A_{rms} = \sqrt{\frac{\sum_{i=1}^{n}(SpO_{2i} - S_{\mathrm{R}i})^2}{n}}$$

We will also compute the bias between gold standard, clinical standard and each device under test:

$$B = \frac{\sum_{i=1}^{n}(SpO_{2i} - S_{\mathrm{R}i})}{n}$$

and the precision:

$$s_{res} = \sqrt{\frac{\sum_{i=1}^{n}(SpO_{2i} - SpO_{2fit,i})^2}{(n-2)}}$$

where SpO2fit, is the value of the fitted curve corresponding to the ‘i’th reference value. Simple statistics about the difference among measurements (mean, standard deviation, percentiles, Bland-Altman plots) will be provided for the all the devices under analysis. Identical statistical methods will be applied to assess the agreement between the estimation of the (i) pulse rate from the pulse

For peer review only

oximeters, and of the (ii) heart rate and (iii) respiratory rate from the patch, and the corresponding reference measurements.

The performance of each pulse oximeter in detecting hypoxemia (at each level <=90%) will be assessed by reporting the optimal sensitivity and specificity pair, identified via Receiver-Operating Characteristic curves [7,12].

Descriptive statistics will be also computed per participant, per skin type and with and without movement artefacts.

Outcome analysis
We will analyse the outcomes on all the participants from whom we collected the data. The accuracy will be compared to the respective reference pulse oximeter. Where paired readings exist (device with clinical or gold standard) they will be included in the analysis. Device readings with no paired clinical or gold standard will be excluded from the analysis.

Primary outcome measure – Sensitivity and specificity for detecting hypoxia at each level <=90%): The analysis plan detailed above will provide data on correlation of each device with 'gold standard' arterial measurements, and their accuracy for the detection of hypoxemia: The output of this analysis will allow selection of the devices which correlate most closely to the 'gold standard' measurements, and provide the highest performance in the detection of hypoxemia.

Secondary outcome measure - Correlation of device outputs i.e. HR, RR, PR and SpO_2, with ECG derived HR, capnography derived RR, arterial blood pulse rate and pulse oximetry, respectively, during movement:
The analysis plan detailed above will provide data on correlation of each device with 'gold standard' arterial measurements during protocolised movement tests. The output of this analysis will allow selection of the devices which correlate most closely to the 'gold standard' measurements.

Ethics and Dissemination
This study has received ethical approval by the East of Scotland Research Ethics Service REC 2 (19/ES/0008). The results will be broadly distributed through conference presentations and peer-reviewed publications.

Safety reporting
A serious adverse event (SAE) occurring to a participant should be reported to the Research Ethics Committee (REC) that gave a favourable opinion of the study where in the opinion of the Chief Investigator the event was 'related' (resulted from administration of any of the research procedures) and 'unexpected' in relation to those procedures. Reports of related and unexpected SAEs should be submitted within 15 working days of the Chief Investigator becoming aware of the event, using the Health Regulatory Agency (HRA) report of serious adverse event form. The research team will also request participant's permission to contact the respective GP and report the SAE through the appropriate channels.

Informed Consent and participant withdrawals
Informed consent will be obtained by the lead researcher or a member of the research team (usually a research nurse/physiotherapist) at the start of the study visit (Appendix 3). All those obtaining consent will have received informed consent training as well as Good Clinical Practice training. Each participant has the right to withdraw from the study at any time, without giving a reason and without affecting their career or quality of their future care. If they wish to withdraw from the study,

For peer review only

we will offer to destroy all gathered information. This will be possible up until the point where we de-identify participants' data.

Data Recording and pseudonymisation

All vital signs data will be collected as per each AMD (Appendix 1). Data derived from these devices will be limited to vital signs measurements and associated waveforms. These will be downloaded from the device to a secure, password protected database. No personal identifiable information will be associated with these data. All data held will be associated with a de-identifiable participant number. Where data is uploaded initially to a cloud server (the Wavelet Health wristband), data will be subsequently downloaded by research staff. The download of data does not remove the de-identifiable data from the cloud storage. Access rights to the cloud data will be as per Cloud Privacy license. Participants will be explicitly advised as to the storage of de-identifiable vital signs data and that this data may be kept within the storage facility indefinitely. This will be made clear to participants prior to consent. Other AMD will record data directly to internal devices from which the data can be retrieved and deleted.

Linkage between pseudonym and identifying information will be held in one place, a password protected database on a networked secure server held by the University of Oxford. Access to this database will be limited to research nurses/allied health professionals only and will be destroyed at the end of the study, once all data has been verified. A spreadsheet will be maintained of de-identifiable participant baseline data, such as date of participation. No identifiable data will be held on this spreadsheet. This data will be entered and validated by the study researchers.

Any paper correspondence (such as CRFs and CFs) will be kept in the Kadoorie Centre in an established research area, behind two access-controlled doors and in locked filing cabinets. All documentation will be archived at the end of the project and retained for five years at the off-site secure archive facility (Re-Store) based at Upper Heyford.

Cloud Storage

As these are commercially available systems, de-identifiable data, with no personal identifiers may be transferred to Cloud storage. Where this is the case, this will be discussed with participants before connecting the equipment prior to consent. Data may remain on the storage system even when downloaded by the research team. Access to storage data is as per Cloud licensing agreement. Participants will be explicitly advised as to the storage of de-identifiable vital signs data and that this data may be kept within the storage facility indefinitely. Other AMD will record data directly to internal devices from which the data can be retrieved and deleted. For one device, the Wavelet Health wristband, de-identified data is transmitted to the device manufacturer's cloud-based system before we are able to access and download these data. There is no alternative to this method of transmission for this device.

Participant compensation

Participants will be reimbursed for travel costs incurred, plus appropriate payment in recognition for their time contribution to the study. They will receive vouchers to the monetary value of £20 for completing the pre-screening telephone interview, and then if willing and eligible to participate, £80 for the complete hypoxia exposure visit making a total of £100 for those who complete the study.

PATIENT AND PUBLIC INVOLVEMENT

This study is part of the vHDU project. During Phase 4 (commenced), we will develop a Patient and Public Involvement (PPI) group for ongoing support and feedback. We have attended a number of local Public Engagement Events, where members of the public showed genuine interest in the advances of wearable monitors and were engaged in our vision of a wireless hospital in the future.

For peer review only

References

1 McQuillan P, Pilkington S, Allan A, *et al.* Confidential inquiry into quality of care before admission to intensive care. *BMJ* 1998;**316**:1853–8. doi:10.1136/BMJ.316.7148.1853

2 Prgomet M, Cardona-Morrell M, Nicholson M, *et al.* Vital signs monitoring on general wards: clinical staff perceptions of current practices and the planned introduction of continuous monitoring technology. *Int J Qual Heal Care* 2016;**28**:515–21. doi:10.1093/intqhc/mzw062

3 Jansen JO, Cuthbertson BH. Detecting critical illness outside the ICU: the role of track and trigger systems. *Curr Opin Crit Care* 2010;**16**:184–90. doi:10.1097/MCC.0b013e328338844e

4 Cardona-Morrell M, Prgomet M, Lake R, *et al.* Vital signs monitoring and nurse–patient interaction: A qualitative observational study of hospital practice. *Int J Nurs Stud* 2016;**56**:9–16. doi:10.1016/j.ijnurstu.2015.12.007

5 Weenk M, van Goor H, Frietman B, *et al.* Continuous Monitoring of Vital Signs Using Wearable Devices on the General Ward: Pilot Study. *JMIR mHealth uHealth* 2017;**5**:e91. doi:10.2196/mhealth.7208

6 Appelboom G, Camacho E, Abraham ME, *et al.* Smart wearable body sensors for patient self-assessment and monitoring. *Arch Public Heal* 2014;**72**:28. doi:10.1186/2049-3258-72-28

7 Louie A, Feiner JR, Bickler PE, *et al.* Four Types of Pulse Oximeters Accurately Detect Hypoxia during Low Perfusion and Motion. *Anesthesiology* 2018;**128**:520–30. doi:10.1097/ALN.0000000000002002

8 Chan A-W, Tetzlaff JM, Altman DG, *et al.* SPIRIT 2013 Statement: Defining Standard Protocol Items for Clinical Trials. *Ann Intern Med* 2013;**158**:200. doi:10.7326/0003-4819-158-3-201302050-00583

9 Hutter D, Kingdom J, Jaeggi E. Causes and mechanisms of intrauterine hypoxia and its impact on the fetal cardiovascular system: a review. *Int J Pediatr* 2010;**2010**:401323. doi:10.1155/2010/401323

10 Shenzhen Viatom Technology Co. L. O2 Vibe Wrist Pulse Oximeter Quick Start Guide. Shenzhen, P.R.China: 2016. https://fccid.io/2ADXK-1600/User-Manual/Users-Manual-3119531.pdf

11 Rowland MJ, Ezra M, Winkler A, *et al.* Calcium channel blockade with nimodipine reverses MRI evidence of cerebral oedema following acute hypoxia. *J Cereb Blood Flow Metab* 2017;:0271678X1772662. doi:10.1177/0271678X17726624

12 Kohyama T, Moriyama K, Kanai R, *et al.* Accuracy of Pulse Oximeters in Detecting Hypoxemia in Patients with Chronic Thromboembolic Pulmonary Hypertension. *PLoS One* 2015;**10**:e0126979. doi:10.1371/journal.pone.0126979

Authors' contributions

Authorship is determined in accordance with the ICMJE guidelines:
CA, SV, PP, LT and PW drafted the initial protocol. CA, SV, EK, JE, LY, OG, CR, MR, MP and MS will conduct the study procedures and data acquisition. AS and MH reviewed protocol and will provide medical cover in the study days. CA drafted the manuscript and all authors reviewed and approved it.

The funders have had no role in the study protocol design or the preparation of this manuscript, and will have no role in the collection, management, analysis and interpretation of the data, or the writing of the final report.

Competing interests statement.

PW and LT report significant grants from the National Institute of Health Research (NIHR), UK and the NIHR Biomedical Research Centre, Oxford, during the conduct of the study. PW and LT report

For peer review only

modest grants and personal fees from Sensyne Health, outside the submitted work. PW and LT work part-time for Sensyne Health and hold shares in the company.

For peer review only

Figure legends

Figure 1 - Devices placement example (dominant hand, combination 3)

Figure 2 - Hypoxia study day set up. Legend: 1- Tablets linked with AMD devices (4 Samsung Tab A, each linked with one AMD: AP-20, WristOX2 3150 BLE, CheckMe O2 and VitalPatch®. 1 IPad 4 connected to the Wavelet). 2- Resuscitation trolley and Oxygen. 3- 7% oxygen in nitrogen cylinder. 4- Hypoxicator apparatus. 5- Philips monitor (model MX450) connected to laptop (IX Trend software). 6- Drip stand with the arterial line pressure bag.

For peer review only

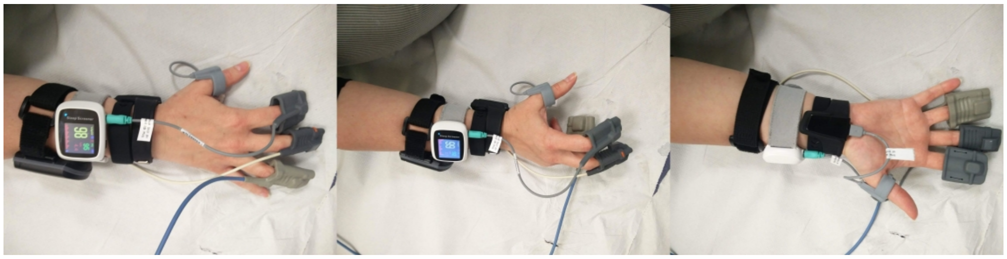

Figure 1: Devices placement example (dominant hand, combination 3)

381x95mm (300 x 300 DPI)

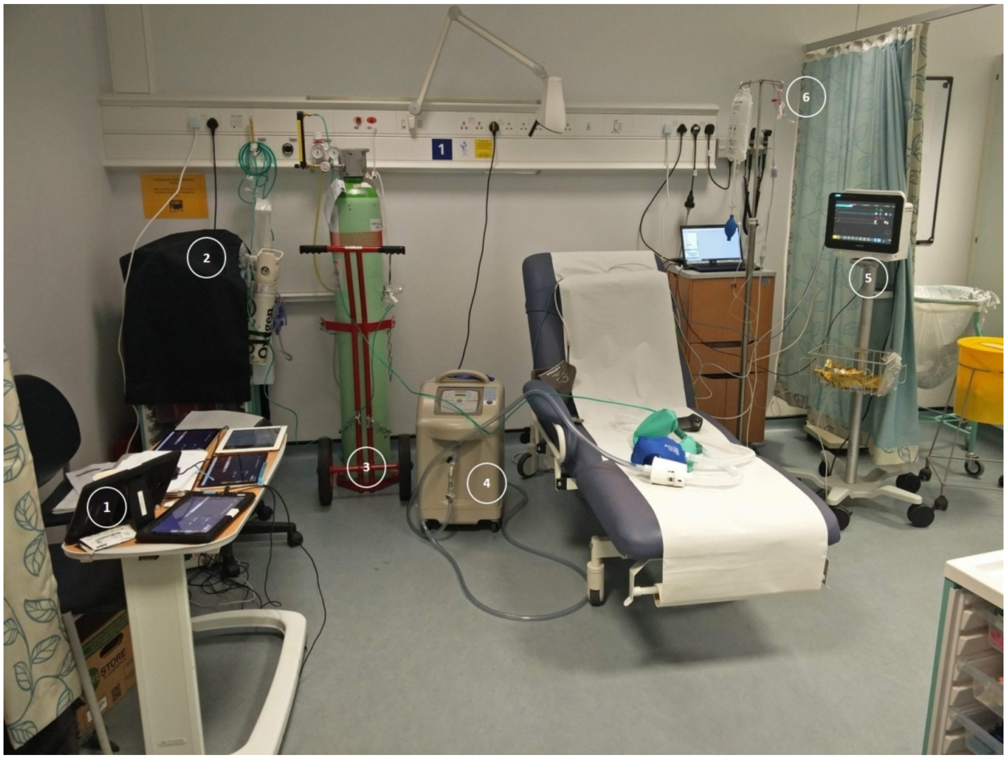

Figure 2: Hypoxia study day set up. Legend: 1- Tablets linked with AMD devices (4 Samsung Tab A, each linked with one AMD: AP-20, WristOX2 3150 BLE, CheckMe O2 and VitalPatch®. 1 IPad 4 connected to the Wavelet). 2- Resuscitation trolley and Oxygen. 3- 7% oxygen in nitrogen cylinder. 4- Hypoxicator apparatus. 5- Philips monitor (model MX450) connected to laptop (IX Trend software). 6- Drip stand with the arterial line pressure bag.

127x95mm (300 x 300 DPI)

Appendix 1: Wearable Ambulatory Monitors Summary

Monitors	Vital signs	Application	Monitoring method	Data storage	
**VitalPatch®**<br><br>**CE marked**	Respiratory rate (rpm),<br>Heart rate (bpm)	Chest worn	1-lead ECG and accelerometer signals are used for the accurate estimation of heart rate and respiratory rate.	Data collected in real time when the device is synchronised via BLE with vHDU app, otherwise the data are recorded in the device's memory, and downloaded afterwards via the vHDU app.	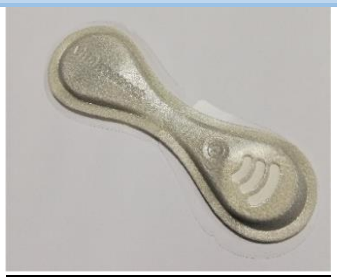
**CheckMe™ O2+**<br><br>**CE Marked**	SpO2 (%),<br>Pulse rate (bpm)	Wrist worn with thumb ring probe.	Transmittance PPG is used to estimate SpO2. The Infrared PPG is used to estimate pulse rate.	Data collected in real time when the device is synchronised via BLE with vHDU app.	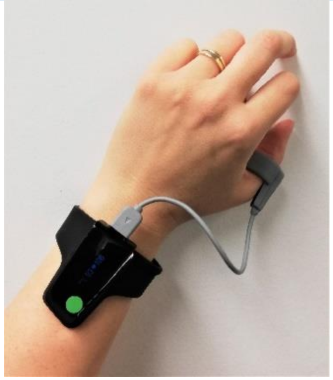

For peer review only

**AP-20®**<br><br>**CE Marked**	SpO2 (%),<br>Pulse rate (bpm),<br>Respiratory rate (rpm).	Wrist worn with finger-tip sensor. Includes Nasal flow sensor.	Transmittance PPG is used to estimate SpO2. The Infrared PPG is used to estimate pulse rate.<br><br>The airflow signal is used to determine the Respiratory Rate	Data collected in real time when the device is synchronised via BLE with vHDU app.<br><br>All data are also stored in the device memory, and downloaded afterwards via a the AP-20® software.	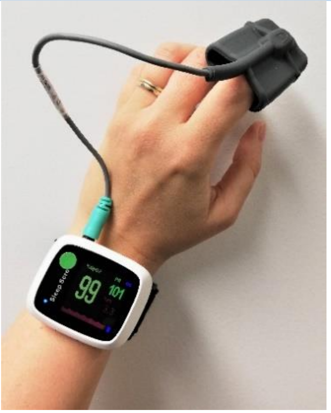
**Wavelet**<br><br>**No regulatory approval at the time of the study**	SpO2 (%),<br>Pulse rate (bpm)	Wrist worn	Reflectance PPG is used to estimate SpO2. The Infrared PPG is used to estimate pulse rate.<br><br>Accelerometer data is used to discard estimations perturbed by moment.	Wavelet onsite App is used to collect the data.<br><br>De-identifiable Data will be transferred to the Cloud storage but data may remain on the storage system even after being downloaded by the research team<br><br>Access to storage data is as per Cloud licensing agreement.	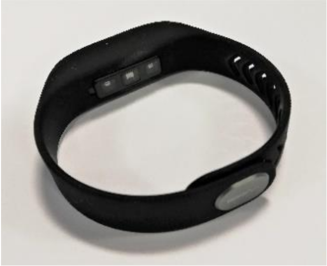
**<u>WristOX2</u> 3150 OEM BLE**<br><br>**FDA Approved**	SpO2 (%),<br>Pulse rate (bpm)	Wrist worn with finger-tip sensor.	Transmittance PPG is used to estimate SpO2. The Infrared PPG is used to estimate pulse rate.	Data collected in real time when the device is synchronised via BLE with vHDU app.	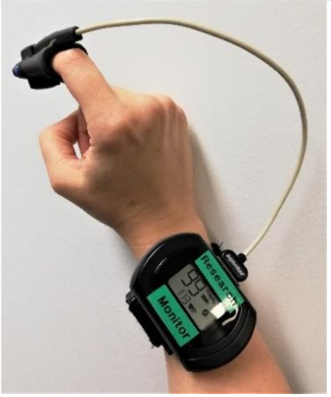

For peer review only

Appendix 2: Functional Movement Testing – Tablet Protocol

Press home
Type in code _ _ _ _
Go to safari (compass icon)
Search 'Google'
Search 'Oxford weather forecast'
Select BBC weather
Scroll across hourly forecast and select 'see more weather for _______'
Scroll across again
Scroll down the page and select and play the BBC South Today weather video.
Press the full screen button in the right bottom corner of the screen. Watch the video then minimise the screen.
Go to the internet search bar and search 'google'
Search 'Amazon'
Select the Amazon website
Search 'Bicycle' and scroll to select a bicycle you like
Add to basket, then navigate back to the search page
Search 'Bicycle Helmet' and again scroll and select one.
Press the back button in the top left corner until back to the safari/google page
Type following sums:
100 x 70 / 45 =
80 + 6 + 95 + 43 + 51 + 15 =

For peer review only

Appendix 3: Model Consent Form

Study Code: *Sub-Study code:* *Participant identification number:*

**V**	**H**	**D**	**U**	—	H	—			

CONSENT FORM

Virtual HDU: Hypoxia Study. Accuracy and validity testing of ambulatory monitoring system.

Name of Researcher: *If you agree, please initial box*

1. I confirm that I have read and understood the information sheet dated 17/JUN/2019 version 4.0 for the above study I have had the opportunity to consider the information, ask questions and have had these answered satisfactorily.	
2. I understand that my participation is voluntary and that I am free to withdraw at any time without giving any reason, without my medical care or legal rights being affected.	
3. I understand who will have access to personal data provided, how the data will be stored and what will happen to the data at the end of the project.	
4. I understand and consent for all required screening tests that will include medical history review, breathing tests, urine pregnancy test (female participants only) and blood sample tests.	
5. I agree to physiological vital sign monitoring with the use of ambulatory monitoring device(s).	
6. I agree to cannulation (insertion of tube in your radial artery) and hypoxic exposure (controlled reduction of oxygen levels) for the duration of the testing phase of the study and understand these procedures and potential (although very rare) complications.	
7. I agree to donate up to 15(teaspoon-sized) blood samples. I consider these samples a gift to the University of Oxford and I understand I will not gain any direct personal or financial benefit from them. I also understand these will be discarded and not retained by the research team after the study visit.	
8. I understand that in some cases the monitoring systems being used require initial upload of vital signs data to their proprietary Cloud storage facility that might be abroad, from which these data are then downloaded. I understand in this case this will be discussed with me beforehand and no identifiable data will be included in this upload.	
9. I understand how to raise a concern and make a complaint.	
**10. I agree to take part in this study.**	

_____________________ _______________ _________________________

Name of Participant *Date* *Signature*

_____________________ _______________ _________________________

Name of Person taking Consent *Date* *Signature*

**1 copy for participant; 1 copy for researcher site file; 1 (original) to be kept in medical notes* (if participant is a patient).

For peer review only

STANDARD PROTOCOL ITEMS: RECOMMENDATIONS FOR INTERVENTIONAL TRIALS

SPIRIT 2013 Checklist: Recommended items to address in a clinical trial protocol and related documents*

Section/item	Item No	Description	Addressed on page number
**Administrative information**			
Title	1	Descriptive title identifying the study design, population, interventions, and, if applicable, trial acronym	1
Trial registration	2a	Trial identifier and registry name. If not yet registered, name of intended registry	2
	2b	All items from the World Health Organization Trial Registration Data Set	Throughout
Protocol version	3	Date and version identifier	2
Funding	4	Sources and types of financial, material, and other support	2
Roles and responsibilities	5a	Names, affiliations, and roles of protocol contributors	1, 12
	5b	Name and contact information for the trial sponsor	2
	5c	Role of study sponsor and funders, if any, in study design; collection, management, analysis, and interpretation of data; writing of the report; and the decision to submit the report for publication, including whether they will have ultimate authority over any of these activities	12
	5d	Composition, roles, and responsibilities of the coordinating centre, steering committee, endpoint adjudication committee, data management team, and other individuals or groups overseeing the trial, if applicable (see Item 21a for data monitoring committee)	N/A

For peer review only - http://bmjopen.bmj.com/site/about/guidelines.xhtml

Introduction

Background and rationale	6a	Description of research question and justification for undertaking the trial, including summary of relevant studies (published and unpublished) examining benefits and harms for each intervention	3
	6b	Explanation for choice of comparators	3
Objectives	7	Specific objectives or hypotheses	3
Trial design	8	Description of trial design including type of trial (eg, parallel group, crossover, factorial, single group), allocation ratio, and framework (eg, superiority, equivalence, noninferiority, exploratory)	3,4

Methods: Participants, interventions, and outcomes

Study setting	9	Description of study settings (eg, community clinic, academic hospital) and list of countries where data will be collected. Reference to where list of study sites can be obtained	7
Eligibility criteria	10	Inclusion and exclusion criteria for participants. If applicable, eligibility criteria for study centres and individuals who will perform the interventions (eg, surgeons, psychotherapists)	4
Interventions	11a	Interventions for each group with sufficient detail to allow replication, including how and when they will be administered	5-7
	11b	Criteria for discontinuing or modifying allocated interventions for a given trial participant (eg, drug dose change in response to harms, participant request, or improving/worsening disease)	5
	11c	Strategies to improve adherence to intervention protocols, and any procedures for monitoring adherence (eg, drug tablet return, laboratory tests)	5-7
	11d	Relevant concomitant care and interventions that are permitted or prohibited during the trial	N/A
Outcomes	12	Primary, secondary, and other outcomes, including the specific measurement variable (eg, systolic blood pressure), analysis metric (eg, change from baseline, final value, time to event), method of aggregation (eg, median, proportion), and time point for each outcome. Explanation of the clinical relevance of chosen efficacy and harm outcomes is strongly recommended	5-7
Participant timeline	13	Time schedule of enrolment, interventions (including any run-ins and washouts), assessments, and visits for participants. A schematic diagram is highly recommended (see Figure)	6,7

For peer review only - http://bmjopen.bmj.com/site/about/guidelines.xhtml

Sample size	14	Estimated number of participants needed to achieve study objectives and how it was determined, including clinical and statistical assumptions supporting any sample size calculations	4
Recruitment	15	Strategies for achieving adequate participant enrolment to reach target sample size	4
**Methods: Assignment of interventions (for controlled trials)**			
Allocation:			
Sequence generation	16a	Method of generating the allocation sequence (eg, computer-generated random numbers), and list of any factors for stratification. To reduce predictability of a random sequence, details of any planned restriction (eg, blocking) should be provided in a separate document that is unavailable to those who enrol participants or assign interventions	N/A
Allocation concealment mechanism	16b	Mechanism of implementing the allocation sequence (eg, central telephone; sequentially numbered, opaque, sealed envelopes), describing any steps to conceal the sequence until interventions are assigned	N/A
Implementation	16c	Who will generate the allocation sequence, who will enrol participants, and who will assign participants to interventions	N/A
Blinding (masking)	17a	Who will be blinded after assignment to interventions (eg, trial participants, care providers, outcome assessors, data analysts), and how	N/A
	17b	If blinded, circumstances under which unblinding is permissible, and procedure for revealing a participant's allocated intervention during the trial	N/A
**Methods: Data collection, management, and analysis**			
Data collection methods	18a	Plans for assessment and collection of outcome, baseline, and other trial data, including any related processes to promote data quality (eg, duplicate measurements, training of assessors) and a description of study instruments (eg, questionnaires, laboratory tests) along with their reliability and validity, if known. Reference to where data collection forms can be found, if not in the protocol	8-9
	18b	Plans to promote participant retention and complete follow-up, including list of any outcome data to be collected for participants who discontinue or deviate from intervention protocols	N/A

For peer review only - http://bmjopen.bmj.com/site/about/guidelines.xhtml

Data management	19	Plans for data entry, coding, security, and storage, including any related processes to promote data quality (eg, double data entry; range checks for data values). Reference to where details of data management procedures can be found, if not in the protocol	10-12
Statistical methods	20a	Statistical methods for analysing primary and secondary outcomes. Reference to where other details of the statistical analysis plan can be found, if not in the protocol	10,11
	20b	Methods for any additional analyses (eg, subgroup and adjusted analyses)	N/A
	20c	Definition of analysis population relating to protocol non-adherence (eg, as randomised analysis), and any statistical methods to handle missing data (eg, multiple imputation)	10
**Methods: Monitoring**			
Data monitoring	21a	Composition of data monitoring committee (DMC); summary of its role and reporting structure; statement of whether it is independent from the sponsor and competing interests; and reference to where further details about its charter can be found, if not in the protocol. Alternatively, an explanation of why a DMC is not needed	N/A
	21b	Description of any interim analyses and stopping guidelines, including who will have access to these interim results and make the final decision to terminate the trial	N/A
Harms	22	Plans for collecting, assessing, reporting, and managing solicited and spontaneously reported adverse events and other unintended effects of trial interventions or trial conduct	11
Auditing	23	Frequency and procedures for auditing trial conduct, if any, and whether the process will be independent from investigators and the sponsor	10
**Ethics and dissemination**			
Research ethics approval	24	Plans for seeking research ethics committee/institutional review board (REC/IRB) approval	2
Protocol amendments	25	Plans for communicating important protocol modifications (eg, changes to eligibility criteria, outcomes, analyses) to relevant parties (eg, investigators, REC/IRBs, trial participants, trial registries, journals, regulators)	2

For peer review only - http://bmjopen.bmj.com/site/about/guidelines.xhtml

Consent or assent	26a	Who will obtain informed consent or assent from potential trial participants or authorised surrogates, and how (see Item 32)	12
	26b	Additional consent provisions for collection and use of participant data and biological specimens in ancillary studies, if applicable	N/A
Confidentiality	27	How personal information about potential and enrolled participants will be collected, shared, and maintained in order to protect confidentiality before, during, and after the trial	12
Declaration of interests	28	Financial and other competing interests for principal investigators for the overall trial and each study site	13
Access to data	29	Statement of who will have access to the final trial dataset, and disclosure of contractual agreements that limit such access for investigators	12
Ancillary and post-trial care	30	Provisions, if any, for ancillary and post-trial care, and for compensation to those who suffer harm from trial participation	12
Dissemination policy	31a	Plans for investigators and sponsor to communicate trial results to participants, healthcare professionals, the public, and other relevant groups (eg, via publication, reporting in results databases, or other data sharing arrangements), including any publication restrictions	2
	31b	Authorship eligibility guidelines and any intended use of professional writers	13,14
	31c	Plans, if any, for granting public access to the full protocol, participant-level dataset, and statistical code	2
**Appendices**			
Informed consent materials	32	Model consent form and other related documentation given to participants and authorised surrogates	
Biological specimens	33	Plans for collection, laboratory evaluation, and storage of biological specimens for genetic or molecular analysis in the current trial and for future use in ancillary studies, if applicable	

*It is strongly recommended that this checklist be read in conjunction with the SPIRIT 2013 Explanation & Elaboration for important clarification on the items. Amendments to the protocol should be tracked and dated. The SPIRIT checklist is copyrighted by the SPIRIT Group under the Creative Commons "Attribution-NonCommercial-NoDerivs 3.0 Unported" license.
